# Concerned issues and controversies in perioperative immunotherapy for resectable non-small cell lung cancer

**DOI:** 10.3389/fimmu.2025.1704226

**Published:** 2025-12-02

**Authors:** Jiang Liu, Qun Ren, Yin Cai, Dadong Chen, Xiang Wu, Wenjing Xu

**Affiliations:** Department of Oncology, Xinghua People’s Hospital Affiliated to Yangzhou University, Xinghua, Jiangsu, China

**Keywords:** non-small cell lung cancer (NSCLC), immune checkpoint inhibitors, perioperative immunotherapy, neoadjuvant therapy, adjuvant therapy

## Abstract

Lung cancer is the most prevalent malignant tumor in China, with the highest incidence and mortality rates. Among the various types of lung cancer, non-small cell lung cancer (NSCLC) accounts for approximately 80% to 85%. Radical surgery is the primary treatment for early-stage NSCLC; however, postoperative recurrence remains a significant clinical challenge. The incorporation of perioperative chemotherapy with surgery has yielded only a modest improvement in the 5-year survival rate, approximately 5%, thereby highlighting the urgent need for more effective systemic treatment alternatives. In recent years, immunotherapeutic drugs, represented by programmed death receptor 1/programmed death ligand 1 (PD-1/PD-L1) monoclonal antibodies, have gradually advanced from later-line therapy to front-line treatment for NSCLC, and have now brought breakthrough progress to perioperative treatment. Multiple phase III immunotherapy clinical trials have demonstrated that both neoadjuvant and adjuvant immunotherapies can significantly enhance the pathological response rate, event-free survival (EFS), and disease-free survival (DFS) in patients with stage II to III NSCLC. Such findings have established new treatment standards aimed at reducing recurrence rates and extending overall survival (OS). Additionally, the potential benefits of the “neoadjuvant plus adjuvant” immunotherapy model have been validated, significantly decreasing the risk of postoperative recurrence in specific patient populations. Future research will continue to explore the efficacy of immunotherapy across different subgroups to maximize clinical benefits while minimizing treatment-related toxicity. Nevertheless, the perioperative application of immunotherapy is accompanied by significant concerns and controversies. This review primarily outlines the latest advancements in perioperative immunotherapy and explores some doubts and controversies encountered in clinical practice, aiming to provide strategies and insights for managing and treating NSCLC in the perioperative setting.

## 1 Introduction

With the growing awareness of lung cancer screening and the widespread use of computed tomography (CT) in high-risk populations, there has been a significant rise in the detection rate of early-stage lung cancer (1). Although surgical intervention remains the preferred treatment modality for lung cancer, the recurrence rate following radical resection in patients diagnosed with early-stage NSCLC continues to be elevated. The 5-year recurrence rates are approximately 10% for stage IA1, 10-15% for stage IA2, 15-20% for stage IA3, 20-30% for stage IB, 30-40% for stage IIA, and 40-55% for stage IIB. Furthermore, only 25% to 30% of NSCLC patients can achieve complete tumor clearance postoperatively, underscoring the necessity of perioperative systemic therapy (2). Over the past two decades, platinum-based adjuvant chemotherapy has been recognized as the standard treatment for patients with stage II-IIIA NSCLC after surgery (3, 4). However, research indicates that, while adjuvant chemotherapy significantly enhances DFS compared to surgical intervention alone, its effect on five-year survival rates is relatively limited, approximately 5% (5–7). Consequently, it is imperative to investigate new adjuvant treatment options to extend survival for these patients.

**Figure 1:**
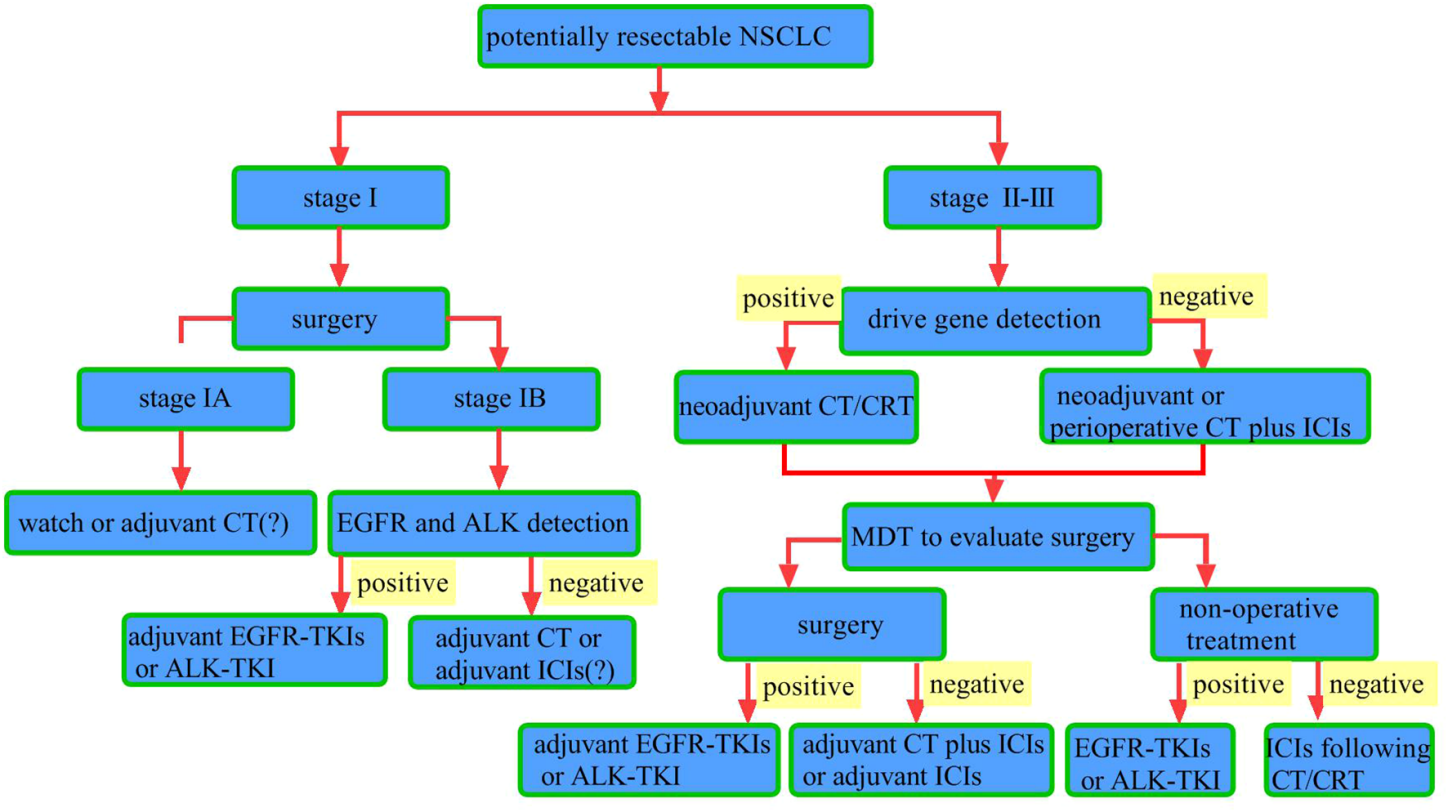
Workflow for potentially resectable NSCLC. Surgical resection is the primary treatment for stage I NSCLC. For patients with pathologic stage I post-surgery, watch-and-wait is an option. The role of adjuvant chemotherapy remains controversial. For patients with pathologic stage IB, testing for driver gene mutations such as epidermal growth factor receptor (EGFR) and anaplastic lymphoma kinase (ALK) is recommended. Patients with identified EGFR mutations should receive adjuvant EGFR-tyrosine kinase inhibitors (TKIs) therapy, while those with ALK rearrangements should receive adjuvant ALK-TKIs therapy. For those without driver gene mutations, adjuvant chemotherapy or immunotherapy may be considered. In stage II–III NSCLC patients with driver gene mutations, chemotherapy (CT) or chemoradiotherapy (CRT) is indicated. For those without driver gene mutations, neoadjuvant chemotherapy combined with immune checkpoint inhibitors (ICIs) is recommended, followed by multidisciplinary team (MDT) evaluation to determine surgical eligibility. For patients deemed eligible for surgery, postoperative driver gene testing should be conducted. Those with driver gene mutations should receive adjuvant targeted therapy, while those without driver gene mutations should receive adjuvant immunotherapy and chemotherapy. For patients with driver gene-positive tumors who are not candidates for surgery, targeted therapy is recommended. For those with driver gene-negative tumors who are inoperable, chemoradiotherapy followed by maintenance immunotherapy is recommended.

In recent years, the advent of immune checkpoint inhibitors (ICIs) has transformed cancer treatment model. By blocking inhibitory signals that suppress T lymphocyte activity, ICIs enhance antitumor immunity (8). As a result, the standard treatment paradigm for NSCLC has evolved, with immunotherapy-based regimens increasingly integrated into both first-line and second-line treatment settings (9–21). For resectable NSCLC, significant breakthroughs in neoadjuvant (22, 23) and adjuvant (24) immunotherapies occurred between 2021 and 2022. In 2023, several phase III studies (25–27) on perioperative (neoadjuvant plus adjuvant) immunotherapy yielded favorable outcomes, establishing this approach as a new standard for reducing recurrence and prolonging survival in patients with resectable NSCLC. Based on the findings of the Neotorch study (28), in January 2024, Toripalimab became the first immunotherapy agent in China to receive approval for perioperative use in NSCLC, marking the commencement of a new era of perioperative immunotherapy for patients with resectable NSCLC in China. Data from key phase III clinical trials incorporating neoadjuvant, adjuvant, and perioperative immunotherapy strategies outline the current treatment landscape for early-stage NSCLC without epidermal growth factor receptor (EGFR) and anaplastic lymphoma kinase (ALK) alterations, as summarized in **Table 1**. However, in the real world, the application of immunotherapy in the perioperative management of NSCLC remains fraught with numerous challenges and controversies. This review primarily explores the common clinical issues and debates surrounding perioperative immunotherapy for NSCLC, aiming to assist clinicians in making better-informed therapeutic decisions. A simplified workflow for potentially clinical resectable NSCLC is revealed in **Figure 1**.

**Table 1:** Significant phase III clinical trials of neoadjuvant, perioperative and adjuvant immunotherapy for resectable NSCLC.

Trial (Ref)	n(stage)	Study arms	Primary endpoints	mEFS (months) (HR; 95% CI)	mOS (months) (HR; 95% CI)	pCR rate (%)	MPR rate (%)
CheckMate 816 (22, 23)	358(Stage IB-IIIA)	Nivo + CT vs. CT	EFS, pCR	43.8 vs. 18.4 HR=0.66 (CI: 0.47-0.90)	NR HR=0.71 (CI:0.47-1.07)	24 vs. 2.2 OR: 13.94 (CI:3.49-55.75)	36.9 vs. 8.9 OR: 5.70 (CI:3.16-10.26)
AEGEAN (26)	802(Stage II–IIIB [N2 node])	CT + Durv →Durv vs. CT→placebo	EFS, pCR	NR vs. 25.3 HR= 0.73 (CI: 0.54–0.98)	NR	17.9 vs. 4.9 Difference:13.0 (CI: 7.1-19.5)	34.2 vs. 14.1 Difference:20.1 (CI: 11.8-28.3)
KEYNOTE 671 (25, 31)	786(Stage II–IIIB [N2 node])	CT +Pembro →Pembro vs. CT→placebo	EFS, OS	47.2 vs. 18.3 HR:=0.59 (CI: 0.48-0.72)	NR vs. 52.4 HR= 0.72 (CI:0.56-0.93)	18.1 vs. 4.1 Difference:14.2 (CI: 10.1-18.7)	30.2 vs. 11 Difference:19.2 (CI: 13.9-24.7)
CheckMate 77T (27, 30)	461(Stage IIA–IIIB)	Nivo + CT→ Nivo vs. CT→placebo	EFS	NR vs. 18.4 HR:=0.58 (CI: 0.42-0.81)	NR	25.3 vs. 4.7 OR=6.64 (CI: 3.4-12.97)	35.4 vs. 12.1 OR=4.01 (CI: 2.48-6.49)
NEOTORCH (28)	404 (Stage II–IIIB)	CT + Tori → Tori vs. CT + placebo →placebo	EFS, MPR	NR vs. 15.1 HR: 0.40 (CI: 0.28-0.57)	NE vs. 30.4 HR: 0.62 (CI:0.33-0.76)	24.8 vs. 1 Difference:23.1 (CI: 17.6-29.8)	48.5 vs. 8.4 Difference:40.2 (CI: 32.2-48.1)
RATIONALE315 (29)	453 (Stage II–IIIA)	CT + TIS → TIS vs. CT + placebo →placebo	EFS, MPR	NR for both HR: 0.56 (CI: 0.40-0.79)	NR for both HR: 0.62 (CI:0.39-0.98)	41 vs. 6 Difference: 35 (CI: 28-42)	56 vs. 15 Difference: 41 (CI: 33-49)
IMpower 010 (24, 36)	1005(Stage IB-IIIA)	Atezo vs. BSC	IA-DFS in Stage II–IIIA (PD-L1 ≥ 1%) Stage II–IIIA (PDL1 ≥ 50%) Stage II–IIIA (all PD-L1) -Stage IB-III (all PD-L1)	68.5 vs. 37.3 0.7 (0.55, 0.91) NR vs. 41.1 0.48 (0.32-0.72) 57.4 vs. 40.8 0.83 (0.69–1.00) 65.6 vs. 47.8 0.85 (0.71–1.01)	NR vs. 87.1 0.77 (0.56–1.06) NR vs. 87.1 0.47 (0.28, 0.77) NR vs. NR 0.94 (0.75, 1.19) NR vs. NR 0.97 (0.78, 1.22)	NA	NA
PEARLS/ KEYNOTE 091 (35)	1177(Stage IB-IIIA)	Pembro vs. placebo	DFS in ITT and PDL1 ≥ 50%	ITT:53.6 vs. 43 0.76( 0.63–0.91) PDL1 ≥50%: NR vs. NR 0.82( 0.57–1.18)	Not reported	NA	NA

mEFS, median event-free survival; mDFS, median disease-free survival; mOS, median overall survival; MPR, major pathologic response, pCR, pathological complete response; HR, hazard ratio; CI, confidence interval (all CIs were at least 95% except where indicated); OR, odds ratio; IA-DFS: investigator-assessed disease-free survival; NR, not reached; NE, not estimable; CT, chemotherapy; Nivo, nivolumab; Pembro, pembrolizumab; Durv, durvalumab; Atezo, atezolizumab; TIS, tislelizumab; BSC, best supportive care; NA, not applicable; Stage IB (tumors ≥ 4 cm); with involvement of ≥1 ipsilateral mediastinal lymph node or subcarinal lymph node [N2 node stage]

## 2 Clinical issues in the real world

### 2.1 The optimal population for perioperative immunotherapy

The objective of perioperative therapy for NSCLC is to establish a comprehensive treatment strategy that is both well-tolerated and effective in improving survival rates. When formulating an immunotherapy-based perioperative regimen, clinicians must consider critical factors such as the clinical stage of the disease and the status of driver gene mutations. Phase III clinical trials (22, 27–30) investigating perioperative immunotherapy, including CheckMate 816, Neotorch, CheckMate 77T, and RATIONALE-315, excluded patients with EGFR mutations or ALK rearrangements. Only KEYNOTE-671 (25, 31) and AEGEAN (26) clinical trials permitted the inclusion of a small fraction of these patients. The subgroup analyses conducted according to disease stage revealed noteworthy distinctions in outcomes. In the Neotorch study (28), the analysis for stage III patients indicated a 56% reduction in the risk of disease progression, recurrence, or mortality in the stage IIIA subgroup, and a 70% reduction in the stage IIIB subgroup. The overall EFS benefit of stage III patients was significant, meeting the study’s prespecified endpoint. Other studies involving patients with stages II and III have also demonstrated significant improvements in EFS across the overall population. The RATIONALE-315 trial (29) presented an EFS hazard ratio (HR) of 0.47 (95% confidence interval: 0.26–0.87) for stage II patients, suggesting a clear therapeutic benefit. Furthermore, findings from KEYNOTE-671 (25), AEGEAN (26), and CheckMate 77T (27) clinical trials exhibited trends toward EFS improvement within stage II subgroups, with hazard ratios of 0.65 (95% CI: 0.42–1.01), 0.76 (95% CI: 0.43–1.34), and 0.81 (95% CI: 0.46–1.43), respectively. Notably, the CheckMate 816 clinical trial (22) highlighted that stage IIIA patients derived a greater relative benefit in EFS compared with those in stage IB–II (HR for stage IIIA, 0.54; HR for stage IB–II, 0.87). Current evidence indicates that patients with resectable, driver gene-negative stage II–IIIB NSCLC can derive significant benefits from perioperative immunotherapy, with stage III patients exhibiting more pronounced improvements in EFS compared with stage II patients.

Given that not all eligible patients currently receive neoadjuvant immunotherapy, the decision to pursue this treatment modality should extend beyond stage and driver mutation status. Critical factors include tumor burden and specific high-risk features. For instance, patients presenting with bulky N2 disease or demonstrating clinical characteristics indicative of a high risk for incomplete resection (R1/R2) or early recurrence are frequently prioritized for neoadjuvant immunotherapy. This approach aims to maximize tumor downstaging and eradicate micrometastases at the outset. Moreover, emerging biomarker profiles are increasingly informing this decision-making process. While PD-L1 expression remains a widely utilized marker, higher expression (e.g. TPS ≥50%) generally correlates with a more substantial therapeutic response. Novel biomarkers—such as tumor mutational burden (TMB), circulating tumor DNA (ctDNA) dynamics, and specific immune gene signatures—are under investigation and hold promise for refining patient selection. In short, the prevailing clinical paradigm advocates for neoadjuvant immunotherapy in patients with heightened disease burden (particularly stage III), high-risk clinical features, and favorable biomarker profiles.

### 2.2 The recommended number of cycles of neoadjuvant immunotherapy

Neoadjuvant immunotherapy is designed to reduce tumor stage, enhance the R0 resection rate, and eliminate subclinical micro-metastases, thereby lowering the risk of postoperative recurrence and prolonging survival for patients with resectable NSCLC. However, a short-course regimen may be inadequate to elicit a potent immune response, while extended treatment duration may lead to disease progression or treatment-related adverse events, potentially resulting in missed surgical opportunities. Consequently, identifying the optimal treatment duration is a critical consideration.

Findings from the neoSCORE trial (32) indicate that three cycles of neoadjuvant immunotherapy combined with chemotherapy increased the major pathological response (MPR) rate by 14.5% compared with two cycles, demonstrating good tolerability and suggesting improved postoperative outcomes with three cycles. Current phase III clinical trials on perioperative immunotherapy, such as Neotorch (28) and CheckMate 816 (22), were structured around three cycles of neoadjuvant therapy, while clinical trials including CheckMate 77T (27), KEYNOTE-671 (25), RATIONALE-315 (29), and AEGEAN (26) permitted up to four cycles. Results from these trials have demonstrated that 3 to 4 cycles of neoadjuvant chemo-immunotherapy lead to significant improvements in pathological complete response (pCR), MPR, and EFS compared with neoadjuvant chemotherapy alone. Notably, at the 2024 European Lung Cancer Congress, the CheckMate 77T trial (33) reported outcomes for 158 patients who completed four cycles compared to 20 participants who received fewer than four cycles due to adverse events or disease progression. Among patients who underwent surgery, pCR rates were 32.3% for those completing four cycles and 35.0% for those receiving fewer than four cycles. The MPR rates were 46.2% and 40.0%, respectively, indicating comparable outcomes between the groups. Additionally, CheckMate 816 (22), which also investigated neoadjuvant nivolumab combined with chemotherapy and was designed for three cycles, reported pCR and MPR rates of 24.0% and 36.9%, respectively—similar to the 25.3% pCR and 35.4% MPR observed in CheckMate 77T trial (30).

In conclusion, based on current data regarding EFS, pCR, and MPR, conclusive evidence to determine the optimal number of neoadjuvant immunotherapy cycles remains elusive, and inherent limitations exist in cross-trial comparisons. According to existing phase III data, it is recommended to administer 3 to 4 cycles of neoadjuvant therapy. The specific number of cycles should be tailored according to the particular drug used, the clinical context, and the surgical plan to ensure both efficacy and minimization of risks associated with disease progression or treatment-related adverse events that could delay or prevent surgery.

Upon completion of the planned neoadjuvant therapy, a thorough preoperative re-evaluation is essential. This assessment aims to accurately re-stage the disease, objectively evaluate tumor response and resectability, and identify patients unlikely to benefit from surgery. For patients deemed ineligible for surgery, subsequent management must be tailored to the specific reason for this decision. (1) In cases of disease progression (PD): These patients typically exhibit primary resistance to the initial chemo-immunotherapy. Management should therefore transition to that for metastatic NSCLC, including biomarker testing for driver mutations if not previously done. Additionally, second-line systemic therapy options, possibly combined with local palliative radiotherapy for symptom management, should be considered. (2) If surgery is inadvisable due to treatment-related adverse events but the disease is controlled: For patients who experience complications such as immune-related pneumonitis or myocarditis, yet whose disease remains controlled, the primary focus must be on the effective management of these adverse effects. Once toxicity is adequately addressed and the patient’s clinical condition allows, surgical feasibility should be reassessed. If the surgical window is deemed lost, alternative local curative-intent interventions, such as definitive radiotherapy, may warrant exploration. (3) When new or worsening non-oncological comorbidities preclude surgery: The focus should be on optimizing management of these conditions. A multidisciplinary team (MDT) should concurrently evaluate the potential role of non-surgical local modalities, including definitive radiotherapy, considering that the oncological disease is controlled.

### 2.3 The effect of neoadjuvant immunotherapy on surgery

Based on surgical outcome data derived from current Phase III clinical trials (22, 25–29) on perioperative immunotherapy models, although adverse reactions during the neoadjuvant treatment phase may potentially delay surgery or increase surgical complexity, the proportion of surgeries cancelled due to adverse events across various Phase III studies remains relatively low, ranging from approximately 1.1% to 6.3%. These treatment-related adverse events primarily encompass the typical spectrum of reactions associated with ICIs, including immune-related adverse events (such as rash, colitis, hepatitis, pneumonitis, and endocrinopathies like thyroid dysfunction), as well as systemic symptoms (e.g., fatigue, pyrexia) and side effects from combination chemotherapy (such as myelosuppression, nausea, and vomiting). When comparing experimental groups with control groups, there were no significant differences identified in terms of the proportion of patients undergoing radical surgery, rates of R0 resection, delays in surgery, surgery-related adverse events (predominantly anemia, pain, wound complications, and pneumonia), duration of postoperative hospital stay, or 30-day and 90-day perioperative mortality rates. Moreover, a stratified analysis of the CheckMate-816 study (22), which focused on stages IB-II and IIIA, indicated that patients with stage IIIA disease demonstrated greater improvements in the rates of minimally invasive surgeries, procedure complexity, and median operative time following neoadjuvant immunotherapy compared with those receiving traditional chemotherapy. Overall, neoadjuvant immunotherapy did not significantly increase surgical difficulty or the incidence of perioperative complications compared with neoadjuvant chemotherapy.

### 2.4 The optimal interval between neoadjuvant immunotherapy and surgery

In current phase III clinical trials (22, 26–29) investigating perioperative immunotherapy regimens, the interval between the last neoadjuvant treatment and surgery is predominantly established at 4 to 6 weeks. An interval that exceeds 6 weeks is classified as a surgical delay and is documented as a surgery-related metric. The KEYNOTE-671 trial (25) has a distinct design in that for patients who receive fewer than four cycles of neoadjuvant therapy, the interval between the last treatment and surgery can be extended to up to 8 weeks. Furthermore, this study permits a maximum interval of 20 weeks from the first treatment cycle to surgery. A previous analysis utilizing the National Cancer Database (NCDB) indicated that delays in surgery beyond 6 weeks following neoadjuvant therapy significantly compromise overall survival (34). Consequently, the majority of current clinical trials involving neoadjuvant therapy adopt a 4- to 6-week interval between treatment and surgery, allowing clinicians the flexibility to adjust the timing of surgery within this window based on specific clinical circumstances.

### 2.5 The optimal interval between surgery and adjuvant immunotherapy

Current evidence derived from Phase III clinical trials focusing on the adjuvant immunotherapy-only approach is primarily based on findings from the KEYNOTE-091 (35) and IMpower010 (24, 36) studies. In the KEYNOTE-091 trial (35), the administration of adjuvant chemotherapy was not mandatory. Accordingly, for patients who did not undergo adjuvant chemotherapy, adjuvant immunotherapy was initiated within 12 weeks following surgical intervention. For those who received chemotherapy, a maximum of four cycles was to be completed within 12 weeks post-surgery, after which adjuvant immunotherapy commenced between 3 and 12 weeks following the final chemotherapy cycle. In contrast, the IMpower010 trial (24) mandated at least one cycle of adjuvant chemotherapy, with adjuvant immunotherapy initiated within a window of 3 to 8 weeks after the completion of the last chemotherapy cycle. Among patients who received neoadjuvant immunotherapy, the initiation of adjuvant immunotherapy varied across different studies: in RATIONALE-315 (29), administration occurred within 2 to 8 weeks post-surgery; in AEGEAN (26), within 10 weeks post-surgery; in Neotorch (11), within 4 to 8 weeks post-surgery; in KEYNOTE-671 (25), within 4 to 12 weeks post-surgery; and in CheckMate 77T (30), within 90 days following surgery. Based on the aforementioned research, it is recommended that adjuvant immunotherapy be initiated within 12 weeks after surgery as part of perioperative immunotherapy. Nonetheless, clinical practice may necessitate adjustments to this timeline, taking into account the individual circumstances of each patient.

### 2.6 The duration of postoperative adjuvant immunotherapy

For patients who did not receive neoadjuvant immunotherapy in conjunction with chemotherapy, findings from the phase III IMpower010 and KEYNOTE-091 trials indicated that a duration of one year of adjuvant immunotherapy significantly enhanced disease-free survival (DFS) in comparison to placebo in individuals with completely resected (R0) NSCLC (24, 35). Several phase III clinical trials investigating perioperative immunotherapy modalities for resectable NSCLC have demonstrated that the duration of adjuvant immunotherapy typically ranges from 9 to 12 months. The AEGEAN trial (26) administered treatment every 4 weeks for 12 cycles, while CheckMate 77T (27) provided therapy every 4 weeks for one year. Additionally, the RATIONALE-315 trial (29) employed a regimen of every 6 weeks for up to 8 cycles and adopted a 1-year maintenance immunotherapy. In contrast, the Neotorch (28) and KEYNOTE-671 (25) studies implemented a nine-month maintenance approach, delivering treatments every 3 weeks for 13 cycles. Thus, it is recommended that adjuvant immunotherapy following neoadjuvant immunochemotherapy be administered for a period of 9 to 12 months. In comparison to the neoadjuvant-only strategy evaluated in CheckMate-816 (22), multiple perioperative studies have established that the incorporation of adjuvant immunotherapy confers additional benefits for patients who do not achieve a pCR, thereby reducing the risk of disease progression, recurrence, or mortality. However, for patients who achieve pCR after surgery, it remains uncertain whether the intensity of adjuvant therapy can be reduced and whether minimal residual disease (MRD) detection can guide subsequent treatment strategies. Currently, there is a lack of large-scale prospective clinical data to adequately inform these considerations. The recommended duration for adjuvant immunotherapy is between 9 and 12 months based on the existing evidence from both standalone adjuvant immunotherapy and perioperative immunotherapy studies. Presently, there is insufficient research data to assess the efficacy of shorter or extended durations of adjuvant immunotherapy, necessitating further investigation to determine the optimal treatment duration.

### 2.7 Should adjuvant immunotherapy be used in combination with chemotherapy?

A meta-analysis conducted by the Lung Adjuvant Cisplatin Evaluation (LACE) collaborative group has demonstrated that postoperative adjuvant chemotherapy enhances the 5-year survival rate by 5% for patients diagnosed with stage IIB–III NSCLC (5). The findings from the IMpower010 study (24) indicate that, in comparison to the placebo group, postoperative chemotherapy followed by one year of adjuvant immunotherapy significantly improves DFS in R0 stage II–IIIA NSCLC patients with PD-L1 expression on 1% or more of tumor cells (hazard ratio [HR], 0.66). However, in the intention-to-treat (ITT) population, which encompasses stage IB–IIIA NSCLC patients, the prespecified statistical significance boundary was not achieved, resulting in an HR of 0.81 (95% confidence interval [CI]: 0.67–0.99, P = 0.0395). Conversely, the results from the KEYNOTE-091 study (35) revealed that one year of adjuvant immunotherapy significantly improved DFS in stage IB–IIIA NSCLC patients when compared with the placebo group (HR, 0.76). Subgroup analyses revealed HR values of 0.73 for patients who received adjuvant chemotherapy and 1.25 for those who did not, suggesting that sequential adjuvant immunotherapy following postoperative adjuvant chemotherapy may confer additional survival benefits. Based on these findings, it is recommended that stage II–IIIA NSCLC patients who did not undergo neoadjuvant therapy and who can tolerate chemotherapy should receive adjuvant chemotherapy followed by adjuvant immunotherapy. For patients who are unable to tolerate chemotherapy, postoperative adjuvant immunotherapy alone is recommended. In the case of stage IB NSCLC patients who have undergone R0 resection without receiving neoadjuvant therapy, adjuvant chemotherapy is generally not recommended. However, PD-L1-positive patients with high-risk factors—including poorly differentiated tumors (such as micropapillary adenocarcinoma and neuroendocrine tumors, excluding well-differentiated neuroendocrine tumors), visceral pleural invasion, vascular invasion, or intra-alveolar spread —should undergo comprehensive evaluation through a multidisciplinary approach. Patient preferences should be considered in the decision on whether to use postoperative adjuvant chemotherapy followed by immunotherapy. Currently, the advantages of adjuvant immunotherapy for stage IB NSCLC patients remain limited, necessitating further clinical investigations in this domain.

In key clinical studies of neoadjuvant immunotherapy combined with chemotherapy, including CheckMate 816 (22), CheckMate 77T (27), KEYNOTE-671 (25), AEGEAN (26), and RATIONALE-315 (29), postoperative adjuvant chemotherapy was not included. The Neotorch study (28) is distinct in that it incorporated one cycle of postoperative adjuvant chemotherapy as consolidation therapy. Data from various Phase III studies employing perioperative immunotherapy models indicated that the Neotorch study’s “3 + 1+13” treatment model significantly reduced the risk of disease progression, recurrence, and mortality by 60% in resectable stage III NSCLC populations, outperforming studies that administered only postoperative monotherapy (28). This finding suggests that even a single cycle of postoperative chemotherapy can enhance overall treatment efficacy. Overall, for resectable stage II–IIIb NSCLC patients who have received neoadjuvant immunotherapy in combination with chemotherapy, the recommendation is either adjuvant immunotherapy alone or one cycle of adjuvant chemotherapy combined with immunotherapy, followed by maintenance therapy with immunotherapy exclusively. The summary of postoperative adjuvant treatment pathway is shown in **Table 2**.

**Table 2:** Postoperative adjuvant treatment pathway.

Preoperative treatment model	Recommended postoperative adjuvant therapy	Evidence-based medicine
direct surgery ( without neoadjuvant therapy )		
pathological stage II-IIIA	adjuvant chemotherapy → sequential adjuvant immunotherapy	IMpower010, KEYNOTE-091study
pathological stage II-IIIA	adjuvant immunotherapy alone	KEYNOTE-091 study
pathological stage IB ( with high risk factors )	multidisciplinary assessment, decision-making with patients	The evidence is insufficient, but “chemotherapy plus immunotherapy” can be considered.
neoadjuvant immunotherapy combined with chemotherapy	adjuvant immunotherapy alone	CheckMate 816, CheckMate 77T, KEYNOTE-671, AEGEAN and RATIONALE-315 study
neoadjuvant immunotherapy combined with chemotherapy	one cycle of adjuvant chemotherapy plus adjuvant immunotherapy	Neotorch study

### 2.8 The potential role and challenges of radiotherapy in the perioperative treatment of NSCLC

Radiotherapy induces immunological modifications within tumor cells (37, 38) and has the potential to synergize with immunotherapy by facilitating the release of tumor antigens and modulating the tumor microenvironment. This process can elicit enhanced local and systemic immune responses, a phenomenon referred to as the abscopal effect (39, 40). This combination has demonstrated promising outcomes in both preclinical and clinical studies. However, robust clinical evidence supporting the perioperative combination of immunotherapy and radiotherapy in NSCLC remains limited. In the setting of neoadjuvant therapy for stage III-N2 NSCLC, several phase I–II clinical trials of immunotherapy combined with radiotherapy are currently underway. These investigations aim to establish both the efficacy and safety of this therapy in this preoperative treatment setting. Notably, in phase III trials such as KEYNOTE-671, CheckMate-816, and AEGEAN, only a minor subset of patients received postoperative adjuvant radiotherapy. Furthermore, these trials frequently lack detailed published data regarding the subgroups that underwent adjuvant radiotherapy, thereby limiting insights into the impact of this treatment on distant versus local recurrence. Nevertheless, the combination of adjuvant radiotherapy and immunotherapy in the perioperative management of NSCLC raises valid safety concerns, including potential immunosuppressive effects of radiation (41). These effects are influenced by multiple factors, including total radiation dose, the fractionation, overall treatment duration, and the dose to critical organs such as lymph nodes, the spleen, and bones containing bone marrow. Moreover, key questions remain unresolved, such as the optimal timing for radiotherapy, the appropriate dose and fractionation schemes, and patient selection to maximize therapeutic benefits. In short, the application of radiotherapy presents a complex challenge in the rapidly evolving setting of perioperative immunotherapy. Patients at high risk for locoregional failure, such as those with non-R0 resections, may represent the most appropriate candidates for postoperative adjuvant radiotherapy.

### 2.9 Biomarkers for predicting the efficacy or prognosis of perioperative immunotherapy for NSCLC

#### 2.9.1 PD-L1

Based on the findings from phase III clinical trials (22, 24–29, 35) investigating perioperative immunotherapy, patients derive benefits from this treatment irrespective of their PD-L1 expression status, although those who are PD-L1 positive appear to experience more pronounced advantages. Within the subgroup analysis of the CheckMate 77T study (27), it was observed that the pCR rates in the immunotherapy plus chemotherapy cohort were 12.9%, 26.5%, and 51.1% for patients whose PD-L1 expressions were classified as <1%, 1%–49%, and ≥50%, respectively. Similarly, the AEGEAN study (26) reported the corresponding pCR rates of 9.0%, 16.3%, and 27.5% for the immunotherapy plus chemotherapy group. These results indicate a positive correlation between short-term efficacy and PD-L1 expression levels in combination therapy settings. In relation to the impact of PD-L1 expression levels on EFS, multiple studies (22, 25–29) on perioperative immunotherapy, including regimens solely focused on neoadjuvant treatments, demonstrated that the reduction in the risk of disease progression, recurrence, or mortality was significantly greater in populations expressing PD-L1 (≥1%) compared with those with negative PD-L1 expression (<1%). These suggest that PD-L1 expression may serve as a predictive biomarker for both treatment response and survival benefits in patients with resectable NSCLC undergoing perioperative immunotherapy. The collective findings across all perioperative and neoadjuvant immunotherapy studies underscore the role of PD-L1 expression status as a biomarker predictive of the extent of benefit from this treatment.

The IMpower010 phase III adjuvant immunotherapy study (24) revealed that atezolizumab, administered after adjuvant platinum-based chemotherapy in patients with early-stage NSCLC, significantly enhanced DFS in stage II–IIIA NSCLC patients with PD-L1 expression levels ≥1% on tumor cells, in comparison to best supportive care (HR, 0.66, P = 0.0039). In light of these results, the National Medical Products Administration (NMPA) authorized the use of atezolizumab for adjuvant therapy in stage II–IIIA NSCLC patients with PD-L1 expression ≥1% following complete resection and platinum-based chemotherapy. Additionally, the KEYNOTE-091 phase III adjuvant immunotherapy study (35) demonstrated that pembrolizumab significantly improved DFS compared with placebo in completely resected stage IB–IIIA NSCLC, regardless of PD-L1 expression levels (HR, 0.76, P = 0.0014). However, within the subgroup exhibiting high PD-L1 expression (tumor proportion score ≥50%), the DFS benefit associated with pembrolizumab did not achieve statistical significance when compared with placebo (HR, 0.82, P = 0.14). The discrepancy in this subgroup may stem from a limited sample size and inadequate follow-up duration. Thus, additional follow-up is necessary to ascertain whether significant differences in survival endpoints may become evident. The outcomes of both studies further highlight the critical role of PD-L1 testing in the guidance of adjuvant immunotherapy.

#### 2.9.2 ctDNA

ctDNA pertains to extracellular, cell-free fragments of DNA that originate from tumor cells and are disseminated into the bloodstream through mechanisms such as tumor cell apoptosis or active secretion (42). Studies have demonstrated a positive correlation between ctDNA levels— including features such as single-nucleotide variants and mutant allele frequency in plasma—and tumor burden (43, 44). The detection of ctDNA serves as an indication of the persistence of lung cancer, as well as the potential for clinical progression. In recent years, extensive research (45–47) has underscored the importance of ctDNA-based MRD monitoring as a predictor of prognosis and recurrence in NSCLC. A meta-analysis (48) encompassing 21 eligible studies has revealed a significant association between MRD-positive status following radical therapy and both an increased risk of disease recurrence and shortened overall survival (HR, 4.95, P < 0.001; HR, 3.93, P < 0.001). Furthermore, the recurrence rate was significantly lower in ctDNA-negative patients compared with ctDNA-positive populations (HR, 3.73, P < 0.001). Among individuals with persistent MRD negativity, the recurrence rate was observed to be as low as 3.2–3.4%, thereby aiding in the identification of potentially cured populations (49–51). In the context of perioperative immunotherapy, clinical trials (52–55) have demonstrated a strong correlation between the results of ctDNA monitoring and DFS as well as OS in neoadjuvant and adjuvant treatment settings, respectively. Consequently, ctDNA functions as a robust prognostic biomarker.

**Figure 2:**
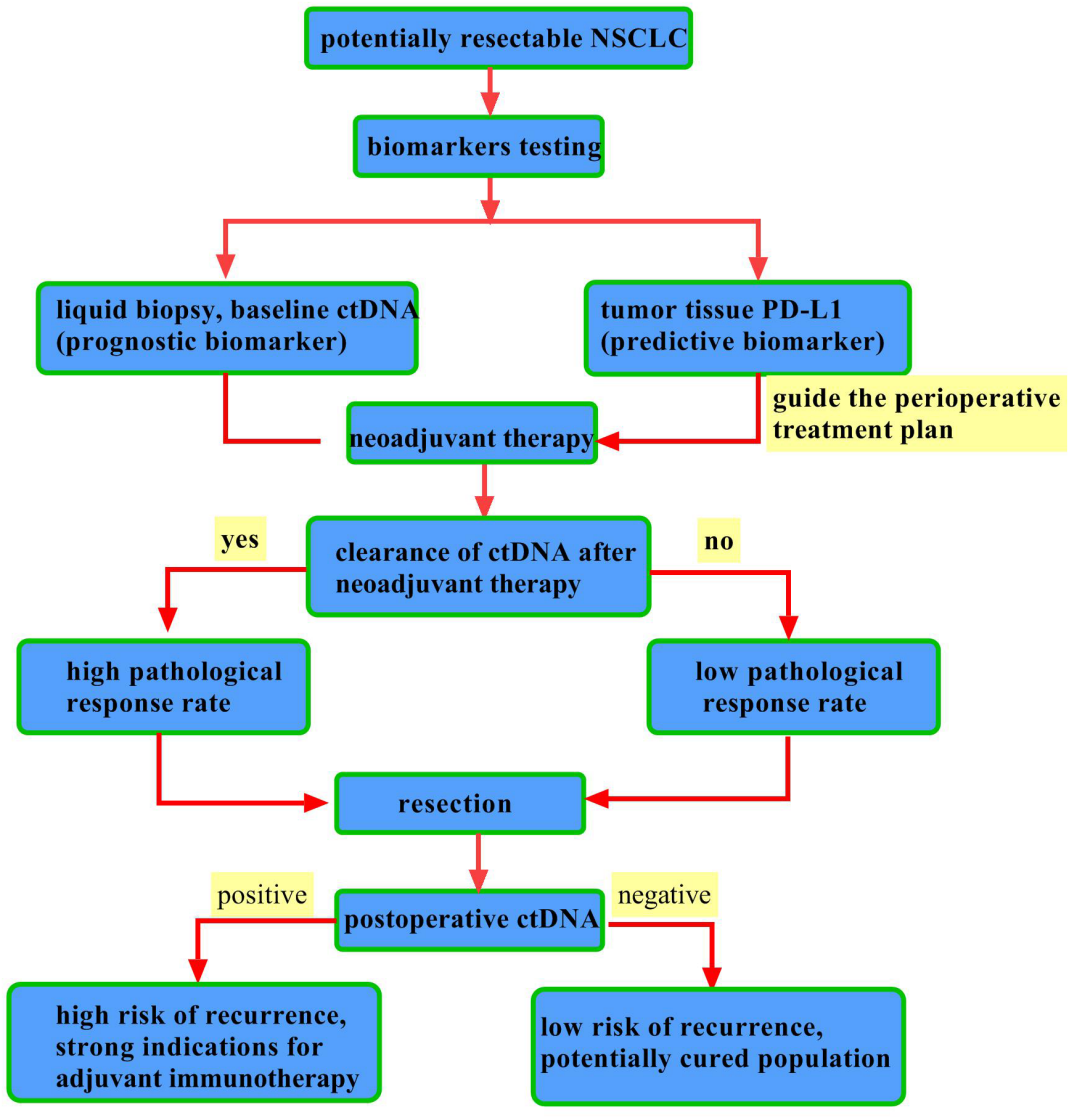
Integrated application of biomarkers in perioperative immunotherapy for NSCLC. This diagram outlines the complementary roles of PD-L1 and ctDNA in perioperative decision-making. PD-L1 is a static, predictive biomarker from a one-time tissue test that guides initial therapy. In contrast, ctDNA is a dynamic, prognostic biomarker tracked via repeated blood tests. Key clinical applications include: ① Post-neoadjuvant: ctDNA clearance indicates a strong early response and predicts better survival. ② Post-surgery: ctDNA positivity defines Minimal Residual Disease (MRD) and high recurrence risk, guiding adjuvant therapy, while sustained negativity suggests a “potentially cured” state with low risk.

The monitoring of ctDNA in peripheral blood has emerged as an innovative strategy for evaluating molecular tumor burden. The levels of ctDNA or the rates of clearance may hold predictive value for efficacy in NSCLC immunotherapy (56–59). Nonetheless, findings across various studies have exhibited an inconsistence, which underscores the necessity for further clinical data and enhanced evidence. The CheckMate 816 trial (22) revealed that among patients with early-stage operable NSCLC, the ctDNA clearance rate was significantly higher in the nivolumab plus chemotherapy group (56%) compared with the chemotherapy-alone group (35%). In both groups, patients achieving ctDNA clearance experienced higher rates of pCR. Likewise, the AEGEAN study reported elevated pCR rates in patients who attained ctDNA clearance (60). A prospective phase II trial (61) demonstrated that preoperative ctDNA clearance was significantly associated with a higher MPR rate compared with those with residual ctDNA (88.9% *vs*. 8.3%, P < 0.001). Conversely, the IMpower010 study (55) indicated that in patients with resectable stage II–IIIA NSCLC, the administration of atezolizumab following chemotherapy improved DFS relative to best supportive care, irrespective of ctDNA status, thus suggesting that ctDNA may not serve as a reliable predictor of treatment response. However, the Imvigor010 study (62) in urothelial cancer found that ctDNA-positive patients experienced significant OS benefits from immunotherapy (HR, 0.59). The observed discrepancies among these studies may be attributed to variations in ctDNA assay platforms and disease-specific factors. Further investigation is warranted to elucidate the predictive value of ctDNA in relation to perioperative immunotherapy. The integrated application of biomarkers in perioperative immunotherapy for NSCLC is revealed in **Figure 2**.

### 2.10 What molecular examinations should be performed before neoadjuvant immunotherapy and after surgery?

The Phase III CheckMate 816 trial (22) demonstrated that, in patients with resectable stage IB–IIIA NSCLC, neoadjuvant immunotherapy significantly improved both EFS (HR = 0.63; P = 0.005) and pCR rate (OR = 13.94; P < 0.001) compared with chemotherapy alone. Similarly, the Phase III Neotorch trial (28) investigated perioperative immunotherapy and demonstrated a significant improvement in both EFS (HR = 0.40; P < 0.001) and MPR rate (between-group difference, 40.2%; P < 0.001) compared with chemotherapy alone in patients with resectable stage IIIA–IIIB NSCLC. In light of these findings, the NMPA has approved nivolumab or toripalimab as neoadjuvant treatment for resectable NSCLC. Both the CheckMate 816 and Neotorch trials exclusively enrolled patients with EGFR/ALK wild-type, thereby highlighting the necessity of conducting tests for EGFR and ALK status before the neoadjuvant or perioperative immunotherapy, which is similar to the CheckMate 77T (27, 30) and RATIONALE-315 (29) studies. In contrast, the KEYNOTE-671 (25) and AEGEAN (26) trials included 33 and 51 patients with EGFR mutations, respectively, with reported EFS HR of 0.09 and 0.86. The small sample sizes and inconsistent outcomes suggest that the efficacy of perioperative immunotherapy in EGFR-mutant NSCLC remains uncertain. Notably, the KEYNOTE-671 trial included 21 patients with ALK rearrangements, but no efficacy data were made available for this cohort.

The CheckMate 77T (27) and AEGEAN (26) trails revealed a positive correlation between pCR rates and levels of PD-L1 expression in the arms combining immunotherapy with chemotherapy. Regarding the relationship between PD-L1 expression and EFS benefit, a consistent reduction in the risk of disease progression, recurrence, or mortality was observed across all perioperative immunotherapy studies (22, 25–29) for PD-L1–positive subgroups (expression ≥1%). This indicates that PD-L1 expression may serve as a promising biomarker for predicting short-term efficacy and survival benefits associated with perioperative immunotherapy in resectable NSCLC patients.

The Phase III ADAURA trial (63) provided compelling evidence that osimertinib significantly enhances DFS in patients with stage IB–IIIA NSCLC harboring sensitive EGFR mutations post-surgery (HR, 0.20, P < 0.001). Another Phase III study (64), ALINA, found that alectinib markedly improved DFS compared with platinum-based chemotherapy in surgically resected stage IB–IIIA NSCLC patients with ALK fusions (HR, 0.24, P < 0.001). These findings underscore the critical role of EGFR and ALK testing in directing adjuvant targeted therapy. Presently, there is a lack of reported Phase III data concerning adjuvant targeted therapies for other driver gene mutations. The Phase III IMpower010 trial (24) demonstrated that atezolizumab significantly improved DFS compared with best supportive care in patients with stage II–IIIA NSCLC whose tumor cells expressed PD-L1 at a level of 1% or greater (TC ≥1%) following resection and chemotherapy (HR, 0.66, P = 0.0039) (17). The NMPA has approved atezolizumab as an adjuvant treatment for stage II–IIIA NSCLC characterized by PD-L1 expression of 1% or greater. In addition, the KEYNOTE-091 trial (35) presented evidence that pembrolizumab significantly improved DFS in completely resected stage IB–IIIA NSCLC, irrespective of PD-L1 expression levels (HR, 0.76, P = 0.0014). Nonetheless, for the subgroup with high PD-L1 expression (TPS ≥ 50%), the DFS advantage associated with pembrolizumab did not reach statistical significance (HR, 0.82; p = 0.14) (18). This unexpected result may be attributable to the limited sample size in this subgroup and insufficient follow-up duration, indicating the necessity for longer-term data to ascertain the emergence of statistically significant differences in survival endpoints. The findings from both studies emphasize the importance of PD-L1 testing in guiding clinical decisions regarding adjuvant immunotherapy.

### 2.11 How to accurately interpret pCR/MPR?

The pathological evaluation of tumor response following neoadjuvant therapy is primarily concerned with determining whether an MPR or a pCR has been achieved. This evaluation is critical for predicting long-term survival and informing treatment strategies for NSCLC. Multiple international guidelines have been established to standardize the pathological assessment of NSCLC after neoadjuvant therapy (65–67). Prominent among these guidelines are the multidisciplinary pathological evaluation recommendations from the International Association for the Study of Lung Cancer (IASLC) (65) and the immune-related pathological response criteria (irPRC) (66, 68). A pCR is defined as the absence of any viable tumor cells in all examined specimens, which includes regional lymph nodes as well as the primary tumor site. The definition of MPR, however, varies slightly between the two guidelines. According to IASLC criteria (65), MPR is characterized by the presence of no more than 10% residual viable tumor cells within the tumor bed, irrespective of the presence of residual tumor cells in the lymph nodes. Conversely, the irPRC mandates that both the lymph nodes and the primary tumor site must exhibit no more than 10% residual viable tumor cells for a designation of MPR (66, 68). Although the CheckMate 816 trial employed the irPRC criteria to define MPR, recent research findings suggest that the percentage of residual viable tumor cells at the primary tumor site is a significant predictor of EFS (69). Preliminary research conducted domestically suggests that the IASLC criteria outperform the irPRC in terms of predicting EFS (70). Furthermore, there remains a lack of consensus regarding the methods and standards for evaluating lymph node metastases following neoadjuvant therapy. Therefore, the IASLC guideline criteria are currently recommended for defining MPR.

### 2.12 Immune-related adverse events management in perioperative settings

For patients with early-stage resectable NSCLC, neoadjuvant immunotherapy is typically administered over a course of 2 to 4 cycles. Compared with patients with advanced NSCLC, the integration of immunotherapy and chemotherapy within the neoadjuvant framework for early-stage resectable cases is associated with a relatively lower incidence of irAEs. This reduction is particularly notable for grade 3 or higher adverse events and those necessitating drug discontinuation, likely due to the shorter treatment duration and better physical condition of early-stage patients (33). A systematic review and meta-analysis, which synthesized data from 2524 participants across six Phase II/III studies, found no significant difference in the incidence of all-grade treatment-related adverse events (TRAEs) between neoadjuvant or perioperative immunotherapy and neoadjuvant chemotherapy for resectable NSCLC (71). In the postoperative adjuvant setting, the duration of treatment in Phase III clinical trials is currently around 9 to 12 months. Due to this longer exposure to the drug, adjuvant immunotherapy tends to have a higher incidence of adverse events compared with neoadjuvant immunotherapy (24, 35). In current Phase III trials of perioperative immunotherapy, the predominant adverse events reported include fever, fatigue, thyroid dysfunction, rash, pneumonia, and enteritis. No new or unexpected adverse events have been identified in these studies compared with those observed during immunotherapy in advanced stages. Therefore, the management and treatment protocols should be similar to those applied in the advanced NSCLC. During immunotherapy, patients must undergo regular general physical examinations, imaging studies, and assessments of hematological and organ functions. Standard monitoring should incorporate routine hematological tests, thyroid function evaluations, adrenal function assessments, and myocardial enzyme profile analysis, conducted at intervals of every four to six weeks. This systematic approach facilitates the early identification, detection, and prevention of irAEs that may not yet present with clinical symptoms. Given the diversity of adverse events associated with immunotherapy, additional laboratory and diagnostic tests may be warranted based on the individual patient’s condition. A study (72) involving 2,750 lung cancer patients treated with ICIs from 2011 to 2020 revealed that 53% of participants experienced irAEs lasting beyond six months. Among these patients, 18 experienced colitis, 4 had pneumonia, and 3 suffered from neuromuscular irAEs, with symptoms persisting for over one year. Consequently, patients should receive continued monitoring and follow-up for a minimum of one year following the conclusion of immunotherapy.

**Figure 3:**
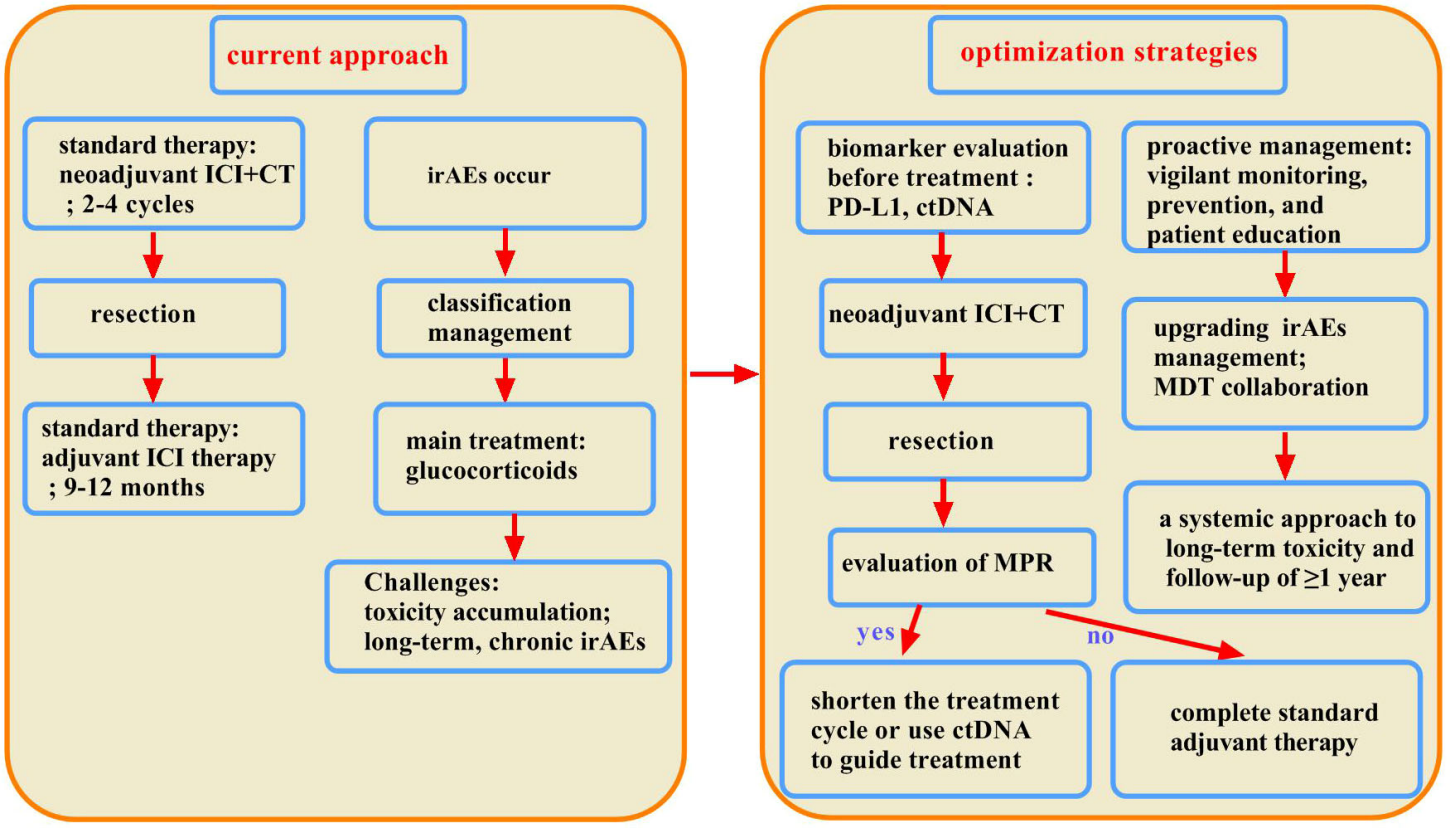
Optimization of perioperative immunotherapy strategies for superior efficacy and reduced toxicity. This chart compares current practice with future strategies for balancing efficacy and toxicity. The current model is reactive, utilizing fixed-duration therapy and passive monitoring, followed by the treatment of immune-related adverse events (irAEs) with corticosteroids. This leads to cumulative toxicity and poor management of chronic or steroid-resistant cases. The future model is proactive and multidimensional, utilizing biomarkers for precise decision-making, focusing on early detection and prevention, and employing advanced interventions such as MDTs and second-line agents for complex irAEs.

Similar to the management of irAEs in advanced NSCLC, corticosteroids should be initiated promptly for patients who develop irAEs during the perioperative period of immunotherapy. Corticosteroid use should adhere to the following principles. i). For irAEs classified as Grade 2 or higher, it is essential to promptly withhold ICIs. Treatment may be recommenced if symptoms and/or laboratory findings improve to Grade 1 or lower. If symptoms persist for more than one week, the initiation of glucocorticoid therapy is advised. ii). For patients experiencing Grade 3–4 irAEs, glucocorticoid treatment should be administered. Glucocorticoid tapering may begin once symptoms have gradually improved to Grade 1 or lower. The total duration of glucocorticoid therapy is typically maintained at 4 to 6 weeks. If no improvement is observed after three days (72 hours) of intravenous glucocorticoids, it is advisable to consider combination therapy or a switch to alternative immunosuppressive agents. Management protocols for specific types of irAEs may diverge from conventional glucocorticoid strategies. For instance, endocrine-related irAEs, such as thyroid dysfunction and hypophysitis, often necessitate the inclusion of hormone replacement therapy alongside standard treatments. In the cases of non-life-threatening irAEs, such as pruritus, glucocorticoid intervention may not be required even at Grade 2 severity. Conversely, for life-threatening irAEs, such as myocarditis, patients presenting with Grade 2 adverse events must immediately discontinue immunotherapy and receive continuous methylprednisolone treatment for three to five days. For myocarditis classified as Grade 3 or higher, immediate high-dose pulse steroid therapy is essential, followed by continued glucocorticoid administration for approximately four weeks after cardiac function returns to baseline levels. In relation to immunotherapy-related pneumonitis, significant attention must be given during the perioperative phase to distinguish it from infections. If infection cannot be excluded, empirical antibiotic therapy should be implemented. For Grade 1 pneumonitis, baseline examinations should be completed while closely monitoring imaging findings, with chest CT and pulmonary function tests repeated at intervals of 3–4 weeks. If progression to Grade 2 occurs, intravenous methylprednisolone should be administered for 48 to 72 hours. If symptoms fail to improve, management should follow the principles for Grade 3 or higher irAEs. Given the significant variations in mechanisms of action and pharmacokinetics among immunosuppressive drugs, agent selection should be individualized based on patient characteristics and irAE type. The optimization strategies of perioperative immunotherapy for superior efficacy and reduced toxicity is showed in **Figure 3**.

### 2.13 Controversies in perioperative immunotherapy for resectable NSCLC

#### 2.13.1 Perioperative treatment for patients with driver gene positive resectable NSCLC

Given the absence of discernible benefits in the mutant subgroups of the KEYNOTE-091 and IMpower010 studies, coupled with the positive outcomes exhibited in two large global phase III trials, ADAURA (63) and ALINA (64), the recommended strategy for patients with resected driver gene-positive (EGFR/ALK-positive) resectable NSCLC is to proceed with surgical intervention followed by adjuvant targeted therapy exclusively. For patients with resectable NSCLC receiving postoperative adjuvant therapy, the approach for those with other driver mutations remains uncertain due to the current lack of robust clinical data.

Significant clinical debate exists regarding the efficacy of neoadjuvant therapy in patients with resectable, driver gene-positive NSCLC. Notably, the CTONG1103 study (73) stands as the first global investigation of perioperative targeted therapy for Stage III EGFR-mutant NSCLC, but did not yield favorable outcomes. Recently, various neoadjuvant studies utilizing third-generation EGFR-targeted agents have been conducted. Findings from two small, prospective Phase II studies (74, 75) indicated that the MPR rate for osimertinib as neoadjuvant therapy was 10% to 15%, while the pCR rate was between 0% and 3%. These results did not fulfill the pre-established statistical criteria and were markedly lower than the pathological response rates previously observed with neoadjuvant immunotherapy in conjunction with chemotherapy in patients who were EGFR and ALK-negative. Within the context of neoadjuvant immunotherapy combined with chemotherapy, several prospective studies have included a limited number of patients with EGFR/ALK mutations. The LCMC3 study (76), which examined neoadjuvant immunotherapy using a single agent, concluded that none of the patients with EGFR/ALK mutations achieved an MPR following two cycles of neoadjuvant atezolizumab, implying limited efficacy from this approach. In the COLUMBIA study (77), where neoadjuvant immunotherapy was combined with chemotherapy, a total of four patients with EGFR mutation were included. Of these, two patients with sensitive EGFR mutations achieved a pCR after two cycles. The NADIM study (78) also included one patient with an EGFR mutation, who similarly achieved a pCR. These preliminary findings suggest that neoadjuvant immunotherapy in combination with chemotherapy may hold potential efficacy in EGFR-mutant NSCLC. Among current Phase III clinical trials investigating perioperative immunotherapy, only the AEGEAN and KEYNOTE-671 studies included small subpopulations of patients with EGFR mutations. The AEGEAN study (79) presented efficacy data for its EGFR mutant subgroup (consisting of 51 patients) at the 2023 World Conference on Lung Cancer (WCLC). These results suggested limited EFS benefits with durvalumab compared with the placebo group (median EFS: 30.8 months *vs*. 19.6 months; HR, 0.86). Furthermore, the MPR rate (7.7% *vs*. 4.0%) and pCR rate (3.8% *vs*. 0%) in the EGFR mutant subgroup were less pronounced than those observed in the modified intention-to-treat population. However, due to the small sample size and the absence of data on factors such as PD-L1 status and EGFR mutation subtypes, these results necessitate careful interpretation. The KEYNOTE-671 study (25) did not disclose pathological response data for its EGFR subgroup; however, subgroup analysis of EFS indicated a significant improvement with neoadjuvant immunotherapy in conjunction with chemotherapy compared with chemotherapy alone (HR, 0.09). It is important to note that the sample size was limited to 33 patients, warranting cautious interpretation. Several prospective studies exploring neoadjuvant immunotherapy regimens in EGFR mutant populations are currently underway. The findings from these studies will further clarify the efficacy benefits associated with neoadjuvant immunotherapy combination strategies for patients with locally advanced, EGFR-mutant NSCLC. For patients with locally advanced, ALK fusion-positive NSCLC, multiple studies (80, 81) have indicated that immunotherapy provides suboptimal efficacy benefits in ALK fusion populations, whereas targeted therapy has demonstrated superior clinical effectiveness. The NAUTIKA1 umbrella study (82) reported at the 2023 WCLC indicated that 66.7% of patients achieved an MPR, and 33.3% achieved a pCR following two cycles of neoadjuvant alectinib. Additionally, a single-center cohort study (83) from China presented at the 2023 American Association for Thoracic Surgery (AATS) annual meeting reported that after a median of three months of neoadjuvant alectinib treatment, 64.7% of patients attained an MPR, and 35.2% achieved a pCR. After a median follow-up of three years, the median PFS had not yet been reached, with no reported mortality events. Consequently, neoadjuvant targeted therapy may represent a superior clinical choice for patients with locally advanced, ALK fusion-positive NSCLC. However, more data are needed to further guide the perioperative treatment strategies for patients with locally advanced NSCLC harboring other driver gene mutations.

#### 2.13.2 Conversion therapy for patients with unresectable locally advanced NSCLC

The definition of unresectable locally advanced NSCLC exhibits significant heterogeneity. In the pre-immunotherapy era, it encompassed certain cases of stage IIIA, IIIB, and all stage IIIC. Specifically, it includes N2 disease with single-station mediastinal lymph nodes possessing a short-axis diameter of ≥3 cm, or multi-station lymph nodes that are matted with a short-axis diameter of ≥2 cm on computed tomography (CT) scans. Additionally, it encompasses T4 tumors that invade adjacent critical structures, such as the esophagus, heart, aorta, or pulmonary veins, as well as those with metastatic nodules in the same lung but within different lobes. All N3 diseases are also classified as unresectable. More patients require a multidisciplinary approach to determine resectability. For patients with unresectable locally advanced NSCLC, findings from the PACIFIC study (84) revealed that consolidative durvalumab administered following concurrent chemo-radiotherapy (CRT) led to a statistically significant improvement in OS when compared with placebo (47.5 months *vs*. 29.1 months, HR, 0.68). Furthermore, the GEMSTONE-301 study (85) illustrated that consolidative immunotherapy significantly enhanced clinical outcomes for patients who underwent sequential chemo-radiotherapy due to their unsuitability for concurrent CRT. Consequently, consolidative immunotherapy following either concurrent or sequential chemo-radiotherapy has been established as the standard treatment for patients with unresectable NSCLC. Nonetheless, it is important to recognize that approximately 5% of patients may experience disease progression during the chemo-radiotherapy phase, and only about one-third of patients in the PACIFIC study achieved long-term survival or long-term DFS. Thus, a subset of patients does not benefit from the standard treatment model in terms of long-term survival.

The CheckMate 816 and NADIM studies have established neoadjuvant immunotherapy as a standard treatment, demonstrating significant improvements in both pathological response rates and survival among patients eligible for surgical resection. Additionally, immunotherapy, when combined with radiotherapy as neoadjuvant treatment for patients with NSCLC, has also yielded significant results. A randomized controlled trial (86) stated that early-stage NSCLC patients treated with neoadjuvant durvalumab in conjunction with stereotactic body radiotherapy (SBRT) exhibited a marked increase in the MPR rate compared with those receiving durvalumab alone (53.3% *vs*. 6.7%), alongside a pCR rate of 26.7%. Importantly, this combination did not lead to a significant increase in treatment-related adverse events (with grade 3–4 adverse event rates at 20% and 17%, respectively), and only one patient (3%) in the combination group experienced surgery delays due to adverse events. Another study (87) indicated that the combination of durvalumab with neoadjuvant stereotactic ablative radiotherapy (SABR) did not prolong surgical time and raised no new safety concerns (with a grade 3–4 adverse event rate of 38%). Therefore, neoadjuvant immunotherapy combined with radiotherapy may offer a chemotherapy-free alternative for patients possessing a high tumor burden and N2 lymph node metastasis who remain candidates for surgical intervention. The substantial short-term efficacy and long-term survival advantages associated with the aforementioned neoadjuvant immunotherapy combined with chemotherapy raise a pertinent question of whether chemo-immunotherapy induction can convert unresectable locally advanced NSCLC into a resectable state. However, robust evidence supporting this approach remains limited. Several small prospective and retrospective studies (88–91) have indicated that among patients initially classified as unresectable due to either large primary tumors, invasion of mediastinal organs, or matted lymph nodes, between 60.7% and 78.6% underwent surgical intervention following 2–3 cycles of combined chemo-immunotherapy. Postoperative MPR rates ranged from 18.8% to 65.5%, with pCR rates exceeding 40%. In a pooled analysis, stage IIIB and IIIC NSCLC patients receiving either chemo-radiotherapy or chemotherapy exhibited relatively favorable clinical outcomes, with 5- and 10-year survival rates for stage IIIB patients at 35% and 27%, respectively, and a median OS of 26 months; stage IIIC patients had 5- and 10-year survival rates of 41% and 29%, respectively (92). Another clinical trial (93) investigating transformative immunotherapy found that, following 3 cycles of induction therapy with a PD-L1/TGF-β bispecific antibody (with or without chemotherapy), 25.2% of patients (27/107) with unresectable locally advanced NSCLC were successfully converted to a resectable status. Conversion rates were 37% for stage IIIA, 44.4% for stage IIIB, and 18.5% for stage IIIC patients; all surgical candidates achieved R0 resection. EFS rates at one year were 74.4% for the converted surgical group and 55.9% for the non-surgical group, with median EFS not reached and averaging 14.9 months, respectively.

The data from these studies indicate that integrating immunotherapy with other treatment modalities may hold the potential to convert certain patients with NSCLC into a resectable state. This finding suggests that immunotherapy may serve as a viable conversion therapy. However, it is crucial to clearly define the patient populations suitable for this conversion. Specifically, patients with N2 disease, which is characterized by single-station lymph nodes with a short-axis diameter of 3 cm or greater, or multi-station matted lymph nodes with a short-axis diameter of 2 cm or greater as indicated by CT scans, may be amenable to conversion therapy. In contrast, the significance of conversion therapy for N3 patients is limited, and direct treatment with the current standard therapy is recommended. For T4 patients exhibiting invasion into vital structures such as the esophagus, heart, aorta, or pulmonary veins, future research on conversion therapy should concentrate on evaluating the risk of local recurrence following immunotherapy and subsequent surgical intervention, even in those achieving a complete response, and whether local treatment is needed upon recurrence. In summary, existing clinical data suggest that neoadjuvant chemo-immunotherapy possesses the capacity to convert a modest proportion of patients with unresectable locally advanced NSCLC to a resectable state, potentially facilitating complete resection. Nonetheless, several pertinent questions persist, including the identification of unresectable NSCLC patients who would benefit from transformative immunotherapy, the determination of an optimal conversion immunotherapy regimen, and the inquiry into whether elevated postoperative pathological response and complete response rates correlate with prolonged survival. These questions warrant further investigation and validation through forthcoming clinical studies. Currently, the standard therapeutic approach for unresectable locally advanced NSCLC remains conversion immunotherapy based on chemo-radiotherapy. For a select group of patients who may achieve conversion via neoadjuvant immunotherapy, it is recommended to participate in the study of conversion immunotherapy following multidisciplinary discussion.

#### 2.13.3 Whether TMB serves as a biomarker for predicting efficacy and prognosis of perioperative immunotherapy in NSCLC

It is widely acknowledged that a high TMB level is associated with increased tumor antigenicity, potentially eliciting a stronger anti-tumor immune response when treated with ICIs. As a result, TMB is often regarded as a predictive biomarker for the efficacy of immunotherapy. However, data on TMB in early-stage NSCLC treated with immunotherapy remain limited. Findings from the CheckMate 159 trial (94) revealed that patients who achieved an MPR had a significantly greater number of mutations compared with those who did not (311 *vs*. 74 mutations, P = 0.01). Similarly, a subgroup analysis of the CheckMate 816 trial (22) demonstrated that a high TMB level (≥12.3 mut/Mb) was linked to improved short-term efficacy, with an MPR rate of 46.2% in the high TMB group compared with 30.6% in the low TMB group, as well as enhanced EFS benefit (HR for high TMB, 0.69 *vs*.HR for low TMB, 0.86). An exploratory analysis of the IMpower010 trial (95) suggested that patients with high TMB derived more significant clinical benefit from adjuvant atezolizumab than those with low TMB. In contrast, the NADIM study (78) found that high TMB (≥10 mutations/Mb) was not associated with improved PFS (HR, 1.67; P = 0.474) or OS (HR, 2.13; P = 0.399). Likewise, the LCMC3 trial and another investigation into neoadjuvant nivolumab combined with ipilimumab found no significant correlation between MPR rate and TMB status (96, 97). Given the limited and conflicting evidence, along with the absence of a standardized TMB cutoff value and uniform detection and analytical methods across laboratories, TMB is not currently recommended for predicting the efficacy and prognosis of perioperative immunotherapy. Further research is essential to better understand the predictive value of TMB in the context of perioperative immunotherapy.

### 2.14 Limitations of current phase III trials

Despite the significant achievements of phase III trials in perioperative immunotherapy, their results must be interpreted and applied with caution due to several inherent limitations. i) Limited data for patients with oncogenic driver mutations. Pivotal phase III trials largely excluded or enrolled very few patients with driver mutations such as EGFR or ALK, leading to a significant gap in evidence for this important subgroup. ii) Potential biases in real-world practice. The rigorous eligibility criteria employed in clinical trials yield a younger patient population who possess a good performance status and demonstrate normal organ function. This population does not accurately reflect the heterogeneity present in real-world clinical settings. Consequently, the efficacy and safety observed in trials may not be directly translatable to older patients, those with poor performance status, or those with significant comorbidities. Real-world evidence is crucial to validate the generalizability of these regimens, especially in underrepresented groups. iii) Incomplete long-term safety profile. Perioperative immunotherapy, particularly the “neoadjuvant plus adjuvant” model, presents unique toxicity management challenges, including delayed immune-related adverse events and potential interactions with surgical complications. The duration of follow-up reported in existing phase III trials remains relatively short; therefore, ongoing monitoring is critical to establish a comprehensive understanding of the long-term safety profile of these interventions.

## 3 Conclusion and outlook

The advent of perioperative immunotherapy for lung cancer signifies a remarkable progression in treatment strategies. Data from clinical studies provide physicians with essential evidence for patient selection and treatment decision-making, enabling thoughtful choices that consider beneficial populations, treatment modalities, and treatment duration. Currently, immunotherapy has become the new standard of treatment for stage II–III NSCLC in the perioperative setting. Nevertheless, various scientific questions remain to be explored. Future research should focus on investigating how novel immunotherapeutic agents can further enhance efficacy, how to identify suitable populations through biomarker-driven selection to maximize treatment benefits, and how to optimize overall perioperative immunotherapy strategies to improve efficacy while minimizing the risks of toxicity.

## References

[1] Krist AH Davidson KW Mangione CM Barry MJ Cabana M Caughey AB . Screening for lung cancer: US preventive services task force recommendation statement. JAMA. (2021) 325:962–70. doi: 10.1001/jama.2021.1117, PMID: 33687470

[2] Le Chevalier T . Adjuvant chemotherapy for resectable non-small-cell lung cancer: where is it going? Ann Oncol. (2010) 21:vii196–198. doi: 10.1093/annonc/mdq376, PMID: 20943614

[3] Goldstraw P Chansky K Crowley J Rami-Porta R Asamura H Eberhardt WE . The IASLC lung cancer staging project: proposals for revision of the TNM stage groupings in the forthcoming (Eighth) edition of the TNM classification for lung cancer. J Thorac Oncol. (2016) 11:39–51. doi: 10.1016/j.jtho.2015.09.009, PMID: 26762738

[4] Kris MG Gaspar LE Chaft JE Kennedy EB Azzoli CG Ellis PM . Adjuvant systemic therapy and adjuvant radiation therapy for stage I to IIIA completely resected non-small-cell lung cancers: american society of clinical oncology/cancer care ontario clinical practice guideline update. J Clin Oncol. (2017) 35:2960–74. doi: 10.1200/JCO.2017.72.4401, PMID: 28437162

[5] Pignon JP Tribodet H Scagliotti GV Douillard JY Shepherd FA Stephens RJ . Lung adjuvant cisplatin evaluation: a pooled analysis by the LACE Collaborative Group. J Clin Oncol. (2008) 26:3552–9. doi: 10.1200/JCO.2007.13.9030, PMID: 18506026

[6] Arriagada R Auperin A Burdett S Higgins JP Johnson DH Le Chevalier T . Adjuvant chemotherapy, with or without postoperative radiotherapy, in operable non-small-cell lung cancer: two meta-analyses of individual patient data. Lancet (London England). (2010) 375:1267–77. doi: 10.1016/S0140-6736(10)60059-1, PMID: 20338627 PMC2853682

[7] Ettinger DS Wood DE Aisner DL Akerley W Bauman J Chirieac LR . Non-small cell lung cancer, version 5.2017, NCCN clinical practice guidelines in oncology. J Natl Compr Cancer Network: JNCCN. (2017) 15:504–35. doi: 10.6004/jnccn.2017.0050, PMID: 28404761

[8] Kang J Zhang C Zhong WZ . Neoadjuvant immunotherapy for non-small cell lung cancer: State of the art. Cancer Commun (London England). (2021) 41:287–302. doi: 10.1002/cac2.12153, PMID: 33689225 PMC8045926

[9] Garassino MC Gadgeel S Speranza G Felip E Esteban E Dómine M . Pembrolizumab plus pemetrexed and platinum in nonsquamous non-small-cell lung cancer: 5-year outcomes from the phase 3 KEYNOTE-189 study. J Clin Oncol. (2023) 41:1992–8. doi: 10.1200/JCO.22.01989, PMID: 36809080 PMC10082311

[10] Novello S Kowalski DM Luft A Gümüş M Vicente D Mazières J . Pembrolizumab plus chemotherapy in squamous non-small-cell lung cancer: 5-year update of the phase III KEYNOTE-407 study. J Clin Oncol. (2023) 41:1999–2006. doi: 10.1200/JCO.22.01990, PMID: 36735893 PMC10082300

[11] Lu S Wang J Yu Y Yu X Hu Y Ma Z . Tislelizumab plus chemotherapy as first-line treatment of locally advanced or metastatic nonsquamous non-small-cell lung cancer (final analysis of RATIONALE-304: a randomized phase III trial). ESMO Open. (2024) 9:103728. doi: 10.1016/j.esmoop.2024.103728, PMID: 39461773 PMC11549519

[12] Wang J Lu S Yu X Hu Y Zhao J Sun M . Tislelizumab plus chemotherapy versus chemotherapy alone as first-line treatment for advanced squamous non-small-cell lung cancer: final analysis of the randomized, phase III RATIONALE-307 trial. ESMO Open. (2024) 9:103727. doi: 10.1016/j.esmoop.2024.103727, PMID: 39461775 PMC11549530

[13] Ren S Chen J Xu X Jiang T Cheng Y Chen G . Camrelizumab plus carboplatin and paclitaxel as first-line treatment for advanced squamous NSCLC (CameL-sq): A phase 3 trial. J Thorac Oncol. (2022) 17:544–57. doi: 10.1016/j.jtho.2021.11.018, PMID: 34923163

[14] Zhang L Wang Z Fang J Yu Q Han B Cang S . Final overall survival data of sintilimab plus pemetrexed and platinum as First-Line treatment for locally advanced or metastatic nonsquamous NSCLC in the Phase 3 ORIENT-11 study. Lung Cancer (Amsterdam Netherlands). (2022) 171:56–60. doi: 10.1016/j.lungcan.2022.07.013, PMID: 35917647

[15] Wang Z Wu L Li B Cheng Y Li X Wang X . Toripalimab plus chemotherapy for patients with treatment-naive advanced non-small-cell lung cancer: A multicenter randomized phase III trial (CHOICE-01). J Clin Oncol. (2023) 41:651–63. doi: 10.1200/JCO.22.00727, PMID: 36206498 PMC9870236

[16] West H McCleod M Hussein M Morabito A Rittmeyer A Conter HJ . Atezolizumab in combination with carboplatin plus nab-paclitaxel chemotherapy compared with chemotherapy alone as first-line treatment for metastatic non-squamous non-small-cell lung cancer (IMpower130): a multicentre, randomised, open-label, phase 3 trial. Lancet Oncol. (2019) 20:924–37. doi: 10.1016/S1470-2045(19)30167-6, PMID: 31122901

[17] Zhou C Wang Z Sun Y Cao L Ma Z Wu R . Sugemalimab versus placebo, in combination with platinum-based chemotherapy, as first-line treatment of metastatic non-small-cell lung cancer (GEMSTONE-302): interim and final analyses of a double-blind, randomised, phase 3 clinical trial. Lancet Oncol. (2022) 23:220–33. doi: 10.1016/S1470-2045(21)00650-1, PMID: 35038432

[18] Zhou C Hu Y Arkania E Kilickap S Ying K Xu F . A global phase 3 study of serplulimab plus chemotherapy as first-line treatment for advanced squamous non-small-cell lung cancer (ASTRUM-004). Cancer Cell. (2024) 42:198–208.e193. doi: 10.1016/j.ccell.2023.12.004, PMID: 38181795

[19] Borghaei H Gettinger S Vokes EE Chow LQM Burgio MA de Castro Carpeno J . Five-year outcomes from the randomized, phase III trials checkMate 017 and 057: nivolumab versus docetaxel in previously treated non-small-cell lung cancer. J Clin Oncol. (2021) 39:723–33. doi: 10.1200/JCO.20.01605, PMID: 33449799 PMC8078445

[20] Zhou C Huang D Fan Y Yu X Liu Y Shu Y . Tislelizumab versus docetaxel in patients with previously treated advanced NSCLC (RATIONALE-303): A phase 3, open-label, randomized controlled trial. J Thorac Oncol. (2023) 18:93–105. doi: 10.1016/j.jtho.2022.09.217, PMID: 36184068

[21] Herbst RS Baas P Kim DW Felip E Pérez-Gracia JL Han JY . Pembrolizumab versus docetaxel for previously treated, PD-L1-positive, advanced non-small-cell lung cancer (KEYNOTE-010): a randomised controlled trial. Lancet (London England). (2016) 387:1540–50. doi: 10.1016/S0140-6736(15)01281-7, PMID: 26712084

[22] Forde PM Spicer J Lu S Provencio M Mitsudomi T Awad MM . Neoadjuvant nivolumab plus chemotherapy in resectable lung cancer. New Engl J Med. (2022) 386:1973–85. doi: 10.1056/NEJMoa2202170, PMID: 35403841 PMC9844511

[23] Spicer J Girard N Provencio M Wang C Mitsudomi T Awad M . Neoadjuvant nivolumab (NIVO)+ chemotherapy (chemo) vs chemo in patients (pts) with resectable NSCLC: 4-year update fromCheckmate 816. J Clin Oncol. (2024) 42:LBA801024. doi: 10.1200/JCO.2024.42.17_suppl.LBA8010

[24] Felip E Altorki N Zhou C Csőszi T Vynnychenko I Goloborodko O . Adjuvant atezolizumab after adjuvant chemotherapy in resected stage IB-IIIA non-small-cell lung cancer (IMpower010): a randomised, multicentre, open-label, phase 3 trial. Lancet (London England). (2021) 398:1344–57. doi: 10.1016/S0140-6736(21)02098-5, PMID: 34555333

[25] Wakelee H Liberman M Kato T Tsuboi M Lee SH Gao S . Perioperative pembrolizumab for early-stage non-small-cell lung cancer. New Engl J Med. (2023) 389:491–503. doi: 10.1056/NEJMoa2302983, PMID: 37272513 PMC11074923

[26] Heymach JV Harpole D Mitsudomi T Taube JM Galffy G Hochmair M . Perioperative durvalumab for resectable non-small-cell lung cancer. New Engl J Med. (2023) 389:1672–84. doi: 10.1056/NEJMoa2304875, PMID: 37870974

[27] Cascone T Awad MM Spicer JD He J Lu S Sepesi B . LBA1 CheckMate 77T: Phase III study comparing neoadjuvant nivolumab (NIVO) plus chemotherapy (chemo) vs neoadjuvant placebo plus chemo followed by surgery and adjuvant NIVO or placebo for previously untreated, resectable stage II–IIIb NSCLC. Ann Oncol. (2023) 34:S1295. doi: 10.1016/j.annonc.2023.10.050

[28] Lu S Zhang W Wu L Wang W Zhang P Fang W . Perioperative toripalimab plus chemotherapy for patients with resectable non-small cell lung cancer: the neotorch randomized clinical trial. JAMA. (2024) 331:201–11. doi: 10.1001/jama.2023.24735, PMID: 38227033 PMC10792477

[29] Yue D Wang W Liu H Chen Q Chen C Liu L . Perioperative tislelizumab plus neoadjuvant chemotherapy for patients with resectable non-small-cell lung cancer (RATIONALE-315): an interim analysis of a randomised clinical trial. Lancet Respir Med. (2025) 13:119–29. doi: 10.1016/S2213-2600(24)00269-8, PMID: 39581197

[30] Cascone T Awad MM Spicer JD He J Lu S Sepesi B . Perioperative nivolumab in resectable lung cancer. New Engl J Med. (2024) 390:1756–69. doi: 10.1056/NEJMoa2311926, PMID: 38749033

[31] Spicer JD Garassino MC Wakelee H Liberman M Kato T Tsuboi M . Neoadjuvant pembrolizumab plus chemotherapy followed by adjuvant pembrolizumab compared with neoadjuvant chemotherapy alone in patients with early-stage non-small-cell lung cancer (KEYNOTE-671): a randomised, double-blind, placebo-controlled, phase 3 trial. Lancet (London England). (2024) 404:1240–52. doi: 10.1016/S0140-6736(24)01756-2, PMID: 39288781 PMC11512588

[32] Shao M Yao J Wang Y Zhao L Li B Li L . Two vs three cycles of neoadjuvant sintilimab plus chemotherapy for resectable non-small-cell lung cancer: neoSCORE trial. Signal transduction Targeted Ther. (2023) 8:146. doi: 10.1038/s41392-023-01355-1, PMID: 37032401 PMC10083171

[33] Awad M Cascone T Spicer J He J Lu S Tanaka F . LBA2 Clinical outcomes with perioperative nivolumab (NIVO) in patients (PTS) with resectable NSCLC from the phase III CheckMate 77Tstudy. ESMO Open. (2024) 9:102985. doi: 10.1016/j.esmoop.2024.102985.34

[34] Gao SJ Corso CD Wang EH Blasberg JD Detterbeck FC Boffa DJ . Timing of surgery after neoadjuvant chemoradiation in locally advanced non-small cell lung cancer. J Thorac Oncol. (2017) 12:314–22. doi: 10.1016/j.jtho.2016.09.122, PMID: 27720827

[35] O'Brien M Paz-Ares L Marreaud S Dafni U Oselin K Havel L . Pembrolizumab versus placebo as adjuvant therapy for completely resected stage IB-IIIA non-small-cell lung cancer (PEARLS/KEYNOTE-091): an interim analysis of a randomised, triple-blind, phase 3 trial. Lancet Oncol. (2022) 23:1274–86. doi: 10.1016/S1470-2045(22)00518-6, PMID: 36108662

[36] Felip E Altorki N Zhou C Vallières E Csoszi T Vynnychenko IO . Five-year survival outcomes with atezolizumab after chemotherapy in resected stage IB-IIIA non-small cell lung cancer (IMpower010): an open-label, randomized, phase III trial. J Clin Oncol. (2025) 43:2343–9. doi: 10.1200/JCO-24-01681, PMID: 40446184

[37] Hu B Jin C Li HB Tong J Ouyang X Cetinbas NM . The DNA-sensing AIM2 inflammasome controls radiation-induced cell death and tissue injury. Sci (New York NY). (2016) 354:765–8. doi: 10.1126/science.aaf7532, PMID: 27846608 PMC5640175

[38] Kumari S Mukherjee S Sinha D Abdisalaam S Krishnan S Asaithamby A . Immunomodulatory effects of radiotherapy. Int J Mol Sci. (2020) 21:8151. doi: 10.3390/ijms21218151, PMID: 33142765 PMC7663574

[39] Kordbacheh T Honeychurch J Blackhall F Faivre-Finn C Illidge T . Radiotherapy and anti-PD-1/PD-L1 combinations in lung cancer: building better translational research platforms. Ann Oncol. (2018) 29:301–10. doi: 10.1093/annonc/mdx790, PMID: 29309540

[40] Hendriks LEL Menis J De Ruysscher DKM Reck M . Combination of immunotherapy and radiotherapy-the next magic step in the management of lung cancer? J Thorac Oncol. (2020) 15:166–9. doi: 10.1016/j.jtho.2019.12.10, PMID: 32127183

[41] Simiele E Dandapani S Han C Wong J Hiniker SM Kovalchuk N . Radiation as an immune modulator: where we are with modern total body irradiation. Semin Radiat Oncol. (2025) 35:67–86. doi: 10.1016/j.semradonc.2024.10.003, PMID: 39672644

[42] Diaz LA Jr. Bardelli A . Liquid biopsies: genotyping circulating tumor DNA. J Clin Oncol. (2014) 32:579–86. doi: 10.1200/JCO.2012.45.2011, PMID: 24449238 PMC4820760

[43] Bergerot PG Hahn AW Bergerot CD Jones J Pal SK . The role of circulating tumor DNA in renal cell carcinoma. Curr Treat Options Oncol. (2018) 19:10. doi: 10.1007/s11864-018-0530-4, PMID: 29464405

[44] Jamal-Hanjani M Wilson GA McGranahan N Birkbak NJ Watkins TBK Veeriah S . Tracking the evolution of non-small-cell lung cancer. New Engl J Med. (2017) 376:2109–21. doi: 10.1056/NEJMoa1616288, PMID: 28445112

[45] Abbosh C Birkbak NJ Wilson GA Jamal-Hanjani M Constantin T Salari R . Phylogenetic ctDNA analysis depicts early-stage lung cancer evolution. Nature. (2017) 545:446–51. doi: 10.1038/nature22364, PMID: 28445469 PMC5812436

[46] Chaudhuri AA Chabon JJ Lovejoy AF Newman AM Stehr H Azad TD . Early detection of molecular residual disease in localized lung cancer by circulating tumor DNA profiling. Cancer Discov. (2017) 7:1394–403. doi: 10.1158/2159-8290.CD-17-0716, PMID: 28899864 PMC5895851

[47] Chen K Zhao H Shi Y Yang F Wang LT Kang G . Perioperative dynamic changes in circulating tumor DNA in patients with lung cancer (DYNAMIC). Clin Cancer Res. (2019) 25:7058–67. doi: 10.1158/1078-0432.CCR-19-1213, PMID: 31439586

[48] Shen H Jin Y Zhao H Wu M Zhang K Wei Z . Potential clinical utility of liquid biopsy in early-stage non-small cell lung cancer. BMC Med. (2022) 20:480. doi: 10.1186/s12916-022-02681-x, PMID: 36514063 PMC9749360

[49] Chen K Yang F Shen H Wang C Li X Chervova O . Individualized tumor-informed circulating tumor DNA analysis for postoperative monitoring of non-small cell lung cancer. Cancer Cell. (2023) 41:1749–1762.e1746. doi: 10.1016/j.ccell.2023.08.010, PMID: 37683638

[50] Zhang JT Liu SY Gao W Liu SM Yan HH Ji L . Longitudinal undetectable molecular residual disease defines potentially cured population in localized non-small cell lung cancer. Cancer Discov. (2022) 12:1690–701. doi: 10.1158/2159-8290.CD-21-1486, PMID: 35543554 PMC9394392

[51] Pan Y Zhang JT Gao X Chen ZY Yan B Tan PX . Dynamic circulating tumor DNA during chemoradiotherapy predicts clinical outcomes for locally advanced non-small cell lung cancer patients. Cancer Cell. (2023) 41:1763–1773.e1764. doi: 10.1016/j.ccell.2023.09.007, PMID: 37816331

[52] Romero A Nadal E Serna R Insa A Campelo MRG Benito C . OA20.02 pre-treatment levels of ctDNA for long-term survival prediction in stage IIIA NSCLC treated with neoadjuvant chemo-immunotherapy. J Thorac Oncol. (2021) 16:S883–4.

[53] Chen D Guo J Huang H Tian L Xie Y Wu Q . Prognostic value of circulating tumor DNA in operable non-small cell lung cancer: a systematic review and reconstructed individual patient-data based meta-analysis. BMC Med. (2023) 21:467. doi: 10.1186/s12916-023-03181-2, PMID: 38012727 PMC10683311

[54] Forde PM Spicer JD Provencio M Mitsudomi T Awad MM Wang C . Overall survival with neoadjuvant nivolumab plus chemotherapy in lung cancer. New Engl J Med. (2025) 393:741–52. doi: 10.1056/NEJMoa2502931, PMID: 40454642

[55] Felip E Srivastava M Reck M Wakelee H Altorki NK Vallieres E . 1O IMpower010: ctDNA status in patients (pts) with resected NSCLC who received adjuvant chemotherapy (chemo) followed by atezolizumab (atezo) or best supportive care (BSC). Immuno-Oncology Technol. (2022) 16:100106. doi: 10.1016/j.iotech.2022.100106

[56] Nabet BY Esfahani MS Moding EJ Hamilton EG Chabon JJ Rizvi H . Noninvasive early identification of therapeutic benefit from immune checkpoint inhibition. Cell. (2020) 183:363–376.e313. doi: 10.1016/j.cell.2020.09.001, PMID: 33007267 PMC7572899

[57] Goldberg SB Narayan A Kole AJ Decker RH Teysir J Carriero NJ . Early assessment of lung cancer immunotherapy response via circulating tumor DNA. Clin Cancer Res. (2018) 24:1872–80. doi: 10.1158/1078-0432.CCR-17-1341, PMID: 29330207 PMC5899677

[58] Ricciuti B Jones G Severgnini M Alessi JV Recondo G Lawrence M . Early plasma circulating tumor DNA (ctDNA) changes predict response to first-line pembrolizumab-based therapy in non-small cell lung cancer (NSCLC). J Immunotherapy Cancer. (2021) 9:e001504. doi: 10.1136/jitc-2020-001504, PMID: 33771889 PMC7996662

[59] Hellmann MD Nabet BY Rizvi H Chaudhuri AA Wells DK Dunphy MPS . Circulating tumor DNA analysis to assess risk of progression after long-term response to PD-(L)1 blockade in NSCLC. Clin Cancer Res. (2020) 26:2849–58. doi: 10.1158/1078-0432.CCR-19-3418, PMID: 32046999 PMC7299781

[60] Reck M Gale D Harpole D Taube JM Mitsudomi T Hochmair MJ . LBA59 Associations of ctDNA clearance and pathological response with neoadjuvant treatment in patients with resectable NSCLC from the phase III AEGEAN trial. Ann Oncol. (2023) 34:S1300. doi: 10.1016/j.annonc.2023.10.055

[61] Xu L Si H Zhuang F Li C Zhang L Zhao Y . Predicting therapeutic response to neoadjuvant immunotherapy based on an integration model in resectable stage IIIA (N2) non-small cell lung cancer. J Thorac Cardiovasc Surg. (2025) 169:242–253.e244. doi: 10.1016/j.jtcvs.2024.05.006, PMID: 38763304

[62] Powles T Assaf ZJ Degaonkar V Grivas P Hussain M Oudard S . Updated overall survival by circulating tumor DNA status from the phase 3 IMvigor010 trial: adjuvant atezolizumab versus observation in muscle-invasive urothelial carcinoma. Eur Urol. (2024) 85:114–22. doi: 10.1016/j.eururo.2023.06.007, PMID: 37500339

[63] Wu YL Tsuboi M He J John T Grohe C Majem M . Osimertinib in resected EGFR-mutated non-small-cell lung cancer. New Engl J Med. (2020) 383:1711–23. doi: 10.1056/NEJMoa2027071, PMID: 32955177

[64] Wu YL Dziadziuszko R Ahn JS Barlesi F Nishio M Lee DH . Alectinib in resected ALK-positive non-small-cell lung cancer. New Engl J Med. (2024) 390:1265–76. doi: 10.1056/NEJMoa2310532, PMID: 38598794

[65] Travis WD Dacic S Wistuba I Sholl L Adusumilli P Bubendorf L . IASLC multidisciplinary recommendations for pathologic assessment of lung cancer resection specimens after neoadjuvant therapy. J Thorac Oncol. (2020) 15:709–40. doi: 10.1016/j.jtho.2020.01.005, PMID: 32004713 PMC8173999

[66] Cottrell TR Thompson ED Forde PM Stein JE Duffield AS Anagnostou V . Pathologic features of response to neoadjuvant anti-PD-1 in resected non-small-cell lung carcinoma: a proposal for quantitative immune-related pathologic response criteria (irPRC). Ann Oncol. (2018) 29:1853–60. doi: 10.1093/annonc/mdy218, PMID: 29982279 PMC6096736

[67] Saqi A Leslie KO Moreira AL Lantuejoul S Shu CA Rizvi NA . Assessing pathologic response in resected lung cancers: current standards, proposal for a novel pathologic response calculator tool, and challenges in practice. JTO Clin Res Rep. (2022) 3:100310. doi: 10.1016/j.jtocrr.2022.100310, PMID: 35498382 PMC9044000

[68] Stein JE Lipson EJ Cottrell TR Forde PM Anders RA Cimino-Mathews A . Pan-tumor pathologic scoring of response to PD-(L)1 blockade. Clin Cancer Res. (2020) 26:545–51. doi: 10.1158/1078-0432.CCR-19-2379, PMID: 31672770 PMC7002263

[69] Deutsch JS Cimino-Mathews A Thompson E Provencio M Forde PM Spicer J . Association between pathologic response and survival after neoadjuvant therapy in lung cancer. Nat Med. (2024) 30:218–28. doi: 10.1038/s41591-023-02660-6, PMID: 37903504 PMC10803255

[70] Sun W Liu X Wang C Jiang Y Lin D . Comparison of different criteria for estimating major pathological response in resectable non-small cell lung cancer treated with neoadjuvant chemoimmunotherapy. Ann Diagn Pathol. (2024) 69:152268. doi: 10.1016/j.anndiagpath.2024.152268, PMID: 38301396

[71] Marinelli D Gallina FT Pannunzio S Di Civita MA Torchia A Giusti R . Surgical and survival outcomes with perioperative or neoadjuvant immune-checkpoint inhibitors combined with platinum-based chemotherapy in resectable NSCLC: A systematic review and meta-analysis of randomised clinical trials. Crit Rev Oncology/Hematology. (2023) 192:104190. doi: 10.1016/j.critrevonc.2023.104190, PMID: 37871779

[72] Luo J Beattie JA Fuentes P Rizvi H Egger JV Kern JA . Beyond steroids: immunosuppressants in steroid-refractory or resistant immune-related adverse events. J Thorac Oncol. (2021) 16:1759–64. doi: 10.1016/j.jtho.2021.06.024, PMID: 34265432 PMC8464489

[73] Zhong WZ Chen KN Chen C Gu CD Wang J Yang XN . Erlotinib versus gemcitabine plus cisplatin as neoadjuvant treatment of stage IIIA-N2 EGFR-mutant non-small-cell lung cancer (EMERGING-CTONG 1103): A randomized phase II study. J Clin Oncol. (2019) 37:2235–45. doi: 10.1200/JCO.19.00075, PMID: 31194613

[74] Lv C Fang W Wu N Jiao W Xu S Ma H . Osimertinib as neoadjuvant therapy in patients with EGFR-mutant resectable stage II-IIIB lung adenocarcinoma (NEOS): A multicenter, single-arm, open-label phase 2b trial. Lung Cancer (Amsterdam Netherlands). (2023) 178:151–6. doi: 10.1016/j.lungcan.2023.02.011, PMID: 36863124

[75] Blakely CM Urisman A Gubens MA Mulvey CK Allen GM Shiboski SC . Neoadjuvant osimertinib for the treatment of stage I-IIIA epidermal growth factor receptor-mutated non-small cell lung cancer: A phase II multicenter study. J Clin Oncol. (2024) 42:3105–14. doi: 10.1200/JCO.24.00071, PMID: 39028931 PMC11379363

[76] Chaft JE Oezkan F Kris MG Bunn PA Wistuba II Kwiatkowski DJ . Neoadjuvant atezolizumab for resectable non-small cell lung cancer: an open-label, single-arm phase II trial. Nat Med. (2022) 28:2155–61. doi: 10.1038/s41591-022-01962-5, PMID: 36097216 PMC9556329

[77] Shu CA Gainor JF Awad MM Chiuzan C Grigg CM Pabani A . Neoadjuvant atezolizumab and chemotherapy in patients with resectable non-small-cell lung cancer: an open-label, multicentre, single-arm, phase 2 trial. Lancet Oncol. (2020) 21:786–95. doi: 10.1016/S1470-2045(20)30140-6, PMID: 32386568

[78] Provencio M Nadal E Insa A García-Campelo MR Casal-Rubio J Dómine M . Neoadjuvant chemotherapy and nivolumab in resectable non-small-cell lung cancer (NADIM): an open-label, multicentre, single-arm, phase 2 trial. Lancet Oncol. (2020) 21:1413–22. doi: 10.1016/S1470-2045(20)30453-8, PMID: 32979984

[79] He J Gao S Reck M Harpole D Mitsudomi T Taube JM . OA12.06 neoadjuvant durvalumab + Chemotherapy followed by adjuvant durvalumab in resectable EGFR-mutated NSCLC (AEGEAN). J Thorac Oncol. (2023) 18:S72–3.

[80] Negrao MV Skoulidis F Montesion M Schulze K Bara I Shen V . Oncogene-specific differences in tumor mutational burden, PD-L1 expression, and outcomes from immunotherapy in non-small cell lung cancer. J Immunotherapy Cancer. (2021) 9:e002891. doi: 10.1136/jitc-2021-002891, PMID: 34376553 PMC8356172

[81] Tian T Li Y Li J Xu H Fan H Zhu J . Immunotherapy for patients with advanced non-small cell lung cancer harboring oncogenic driver alterations other than EGFR: a multicenter real-world analysis. Trans Lung Cancer Res. (2024) 13:861–74. doi: 10.21037/tlcr-24-116, PMID: 38736501 PMC11082706

[82] Lee JM Toloza EM Pass HI Johnson BE Heymach JV Sholl L . P2.01–06 NAUTIKA1 study: preliminary efficacy and safety data with neoadjuvant alectinib in patients with stage IB-III ALK+ NSCLC. J Thorac Oncol. (2023) 18:S297–8.

[83] Zhang C Jiang BY Yan LX . Induction ALK-TKIs for stage III non-small cell lung cancer harboring ALK fusion: a single-center experience with 3-year follow-up. Available online at: https://www.aats.org/resources/abstract.pdf?abstract=1249310:~:text=Induction%20ALK-TKIs%20could%20be%20clinically%20feasible%20in%20stage (Accessed May 6, 2023).

[84] Spigel DR Faivre-Finn C Gray JE Vicente D Planchard D Paz-Ares L . Five-year survival outcomes from the PACIFIC trial: durvalumab after chemoradiotherapy in stage III non-small-cell lung cancer. J Clin Oncol. (2022) 40:1301–11. doi: 10.1200/JCO.21.01308, PMID: 35108059 PMC9015199

[85] Zhou Q Chen M Jiang O Pan Y Hu D Lin Q . Sugemalimab versus placebo after concurrent or sequential chemoradiotherapy in patients with locally advanced, unresectable, stage III non-small-cell lung cancer in China (GEMSTONE-301): interim results of a randomised, double-blind, multicentre, phase 3 trial. Lancet Oncol. (2022) 23:209–19. doi: 10.1016/S1470-2045(21)00630-6, PMID: 35038429

[86] Altorki NK McGraw TE Borczuk AC Saxena A Port JL Stiles BM . Neoadjuvant durvalumab with or without stereotactic body radiotherapy in patients with early-stage non-small-cell lung cancer: a single-centre, randomised phase 2 trial. Lancet Oncol. (2021) 22:824–35. doi: 10.1016/S1470-2045(21)00149-2, PMID: 34015311

[87] Lee B Mynard N Nasar A Villena-Vargas J Chow O Harrison S . Surgical resection after neoadjuvant durvalumab and radiation is feasible and safe in non-small cell lung cancer: Results from a randomized trial. J Thorac Cardiovasc Surg. (2023) 165:327–334.e322. doi: 10.1016/j.jtcvs.2022.07.017, PMID: 36028357

[88] Zeng L Zhou Y Zhang X Xu Q Zhou C Zeng F . Copy number variations mediate major pathological response to induction chemo-immunotherapy in unresectable stage IIIA-IIIB lung cancer. Lung Cancer (Amsterdam Netherlands). (2023) 178:134–42. doi: 10.1016/j.lungcan.2023.02.017, PMID: 36858002

[89] Deng H Liu J Cai X Chen J Rocco G Petersen RH . Radical minimally invasive surgery after immuno-chemotherapy in initially-unresectable stage IIIB non-small cell lung cancer. Ann Surg. (2022) 275:e600–2. doi: 10.1097/SLA.0000000000005233, PMID: 34596079

[90] Xu H Wang W Yin J Song C Li L Sun Z . Efficacy and safety of the PD-1 inhibitor combined with albumin-bound paclitaxel and nedaplatin in preoperative neoadjuvant therapy of unresectable stage III lung squamous cell carcinoma. Drug Design Dev Ther. (2022) 16:4269–77. doi: 10.2147/DDDT.S388777, PMID: 36540715 PMC9760041

[91] Sun C Wang X Xu Y Shao G Chen X Liu Y . Efficiency and safety of neoadjuvant PD-1 inhibitor (sintilimab) combined with chemotherapy in potentially resectable stage IIIA/IIIB non-small cell lung cancer: Neo-Pre-IC, a single-arm phase 2 trial. EClinicalMedicine. (2024) 68:102422. doi: 10.1016/j.eclinm.2024.102422, PMID: 38304743 PMC10831803

[92] Früh M Betticher DC Stupp R Xyrafas A Peters S Ris HB . Multimodal treatment in operable stage III NSCLC: A pooled analysis on long-term results of three SAKK trials (SAKK 16/96, 16/00, and 16/01). J Thorac Oncol. (2019) 14:115–23. doi: 10.1016/j.jtho.2018.09.011, PMID: 30267838

[93] Wu YL Zhou Q Pan Y Yang X Zhao Y Han G . LBA5 A phase II study of neoadjuvant SHR-1701 with or without chemotherapy (chemo) followed by surgery or radiotherapy (RT) in stage III unresectable NSCLC (uNSCLC). Immuno-Oncology Technol. (2022) 16:100361. doi: 10.1016/j.iotech.2022.100361

[94] Forde PM Chaft JE Smith KN Anagnostou V Cottrell TR Hellmann MD . Neoadjuvant PD-1 blockade in resectable lung cancer. New Engl J Med. (2018) 378:1976–86. doi: 10.1056/NEJMoa1716078, PMID: 29658848 PMC6223617

[95] Felip E Srivastava M Reck M Wakelee H Altorki N Vallieres E . MA11.08 IMpower010: exploratory analysis of tumour mutational burden and disease-free survival with adjuvant atezolizumab in NSCLC. J Thorac Oncol. (2023) 18:S139.

[96] Kwiatkowski DJ Rusch VW Chaft JE Johnson BE Nicholas A Wistuba II . Neoadjuvant atezolizumab in resectable non-small cell lung cancer (NSCLC): interim analysis and biomarker data from a multicenter study (LCMC3). J Clin Oncol. (2019) 37:8503. doi: 10.1200/JCO.2019.37.15_suppl.8503.97

[97] Reuss JE Anagnostou V Cottrell TR Smith KN Verde F Zahurak M . Neoadjuvant nivolumab plus ipilimumab in resectable non-small cell lung cancer. J Immunotherapy Cancer. (2020) 8. doi: 10.1136/jitc-2020-001282, PMID: 32929052 PMC7488786

